# Investigation of the Molecular Details of the Interactions of Selenoglycosides and Human Galectin-3

**DOI:** 10.3390/ijms23052494

**Published:** 2022-02-24

**Authors:** Mária Raics, Álex Kálmán Balogh, Chandan Kishor, István Timári, Francisco J. Medrano, Antonio Romero, Rob Marc Go, Helen Blanchard, László Szilágyi, Katalin E. Kövér, Krisztina Fehér

**Affiliations:** 1 Molecular Recognition and Interaction Research Group, Hungarian Academy of Sciences, University of Debrecen, Egyetem tér 1, H-4032 Debrecen, Hungary; raicsmaria@gmail.com (M.R.); balogh.alex@science.unideb.hu (Á.K.B.); 2 School of Chemistry and Molecular Bioscience and Molecular Horizons, University of Wollongong, Wollongong, NSW 2522, Australia; chandan@uow.edu.au; 3 Department of Organic Chemistry, University of Debrecen, Egyetem tér 1, H-4032 Debrecen, Hungary; timari.istvan@science.unideb.hu (I.T.); lszilagyi@unideb.hu (L.S.); 4 Structural and Chemical Biology, Centro de Investigaciones Biolόgicas, Margarita Salas, CSIC Ramiro de Maeztu 9, 28040 Madrid, Spain; fjmedrano@cib.csic.es (F.J.M.); romero@cib.csic.es (A.R.); 5 Institute for Glycomics, Griffith University, Gold Coast, QLD 4222, Australia; rob.go@alumni.griffithuni.edu.au; 6 Department of Inorganic and Analytical Chemistry, University of Debrecen, Egyetem tér 1, H-4032 Debrecen, Hungary

**Keywords:** lectin, galectin-3, selenoglycosides, NMR spectroscopy, fluorescence anisotropy, X-ray crystallography, molecular dynamics

## Abstract

Human galectin-3 (*h*Gal-3) is involved in a variety of biological processes and is implicated in wide range of diseases. As a result, targeting *h*Gal-3 for clinical applications has become an intense area of research. As a step towards the development of novel *h*Gal-3 inhibitors, we describe a study of the binding of two Se-containing *h*Gal-3 inhibitors, specifically that of di(β-D-galactopyranosyl)selenide (SeDG), in which two galactose rings are linked by one Se atom and a di(β-D-galactopyranosyl)diselenide (DSeDG) analogue with a diseleno bond between the two sugar units. The binding affinities of these derivatives to *h*Gal-3 were determined by ^15^N-^1^H HSQC NMR spectroscopy and fluorescence anisotropy titrations in solution, indicating a slight decrease in the strength of interaction for SeDG compared to thiodigalactoside (TDG), a well-known inhibitor of *h*Gal-3, while DSeDG displayed a much weaker interaction strength. NMR and FA measurements showed that both seleno derivatives bind to the canonical S face site of *h*Gal-3 and stack against the conserved W181 residue also confirmed by X-ray crystallography, revealing canonical properties of the interaction. The interaction with DSeDG revealed two distinct binding modes in the crystal structure which are in fast exchange on the NMR time scale in solution, explaining a weaker interaction with *h*Gal-3 than SeDG. Using molecular dynamics simulations, we have found that energetic contributions to the binding enthalpies mainly differ in the electrostatic interactions and in polar solvation terms and are responsible for weaker binding of DSeDG compared to SeDG. Selenium-containing carbohydrate inhibitors of *h*Gal-3 showing canonical binding modes offer the potential of becoming novel hydrolytically stable scaffolds for a new class of *h*Gal-3 inhibitors.

## 1. Introduction

Human galectin-3 (*h*Gal-3), one of the adhesion/growth-regulatory galactose-binding lectins, plays a key role in many different physiological and pathological processes, hence it is involved in a wide variety of diseases such as cancer, inflammation and fibrosis, heart disease and stroke [1,2,3,4]. As one of its main physiological actions, *h*Gal-3 binds β-galactosides of glycoproteins on the cell surface, crosslinks them to form a *h*Gal-3-gylcoprotein lattice [5,6]. This, in turn, increases the residence time of glycoproteins on the cell surface, which prolongs or enhances their activity and prevents protein–protein interactions taking place. In cancer, *h*Gal-3 expression is altered and seems to be associated with tumor-specific T cell inactivation and NK cell activity inhibition [7,8]. *h*Gal-3 is being used as a diagnostic and prognostic biomarker in cancer, heart and kidney disease [9] and drugs targeting the inhibition of *h*Gal-3 are being developed to treat fibrosis [10].

Structurally, *h*Gal-3 [11,12] is composed of two domains [13], an unstructured N terminal collagen-like region with conserved glycine/proline rich repeats that are involved in oligomerization and a C-terminal carbohydrate recognition domain (CRD) that is able to bind sugars. The CRD has a β sandwich-like fold with two antiparallel β sheets, one with a concave sugar binding face (S face, strands S1-S6) consisting of six β strands (S1–S6) and a convex F face formed by five *β*-strands (F1–F5). The sugar binding site is further divided into subsites A-E, whereby subsite C is the canonical β-galactoside binding site, while subsite D interacts with the second, neighboring sugar ring of the natural ligands. The most abundant endogenous ligand of *h*Gal-3, N-acetyllactosamine (NAcLac), comprised of a galactose and N-Acetyl-glucosamine moiety is present in several N- and O-glycans of glycoproteins. The C-4 and C-6 hydroxyl groups and O5 ring oxygen of the galactose unit and the hydroxyl group at the C-3 position of the N-acetyl-glucosamine residue form hydrogen bonds to the sidechain of the protein. The conserved W181 displays a CH-π stacking to the hydrophobic β side of the galactose ring.

**Scheme 1:**
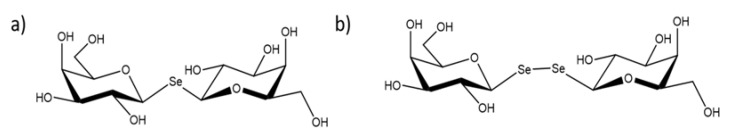
Structure of the tested selenoglycosides (**a**) SeDG and (**b**) DSeDG.

Due to the large potential of *h*Gal-3 as a therapeutic target, a wide range of *h*Gal-3 inhibitors were synthesized and evaluated [14,15]. While major improvements of the binding affinity was mainly achieved by the extension of the core disaccharide to reach out into other subsites, tool compounds and starting scaffolds could be obtained by isosteric substitutions in the core disaccharide, specifically via replacing the oxygen atom in the interglycosidic linkage [16]. Substitution of the oxygen with sulphur yielded a thiodigalactoside (TDG) analogue [17] of the natural ligand showing improved enzymatic/hydrolytic stability. TDG is a potent ligand for adhesion/growth-regulatory ga(lactose-binding) lectins and is used as an immunoadjuvant [18]. TDG displayed a comparable activity to the natural ligands of *h*Gal-3 [17] and was used extensively as a starting scaffold for further derivatives [19,20,21,22,23]. Selenium has been incorporated into carbohydrates to assist in X-ray crystal structure determination using single/multiple wavelength anomalous diffraction techniques [24,25,26] and also in NMR studies [27,28,29]. In our previous work, we proposed replacement of sulphur by selenium in digalactosides as a further bioisosteric substitution [30]. In this report, we investigate the details of the *h*Gal-3 binding of two seleno derivatives, SeDG and DSeDG (Scheme 1a,b, respectively). The molecular details of the recognition of the selenoglycosides by *h*Gal-3 are analyzed using multiple techniques such as solution state NMR spectroscopy, fluorescence anisotropy, X ray crystallography and molecular dynamics (MD) simulations.

## 2. Results and Discussions

### 2.1. Identification of the Binding Site by NMR Chemical-Shift Mapping

Chemical shift mapping via 2D ^15^N-^1^H HSQC of ^15^N-labeled proteins provides residue-specific information allowing mapping of the binding site(s) on the protein [31]. Chemical shift mapping is based on a quantitative analysis of chemical shift perturbation in protein NMR spectra, monitoring the displacements of cross peaks through a series of ^1^H-^15^N HSQC titration spectra. This is best suited for monitoring weak interactions in the μM–mM range when ligand exchange is fast between the free and bound states. In this case, the observed chemical shift is a weighted average of the chemical shifts of the free and complexed protein.

**Figure 1:**
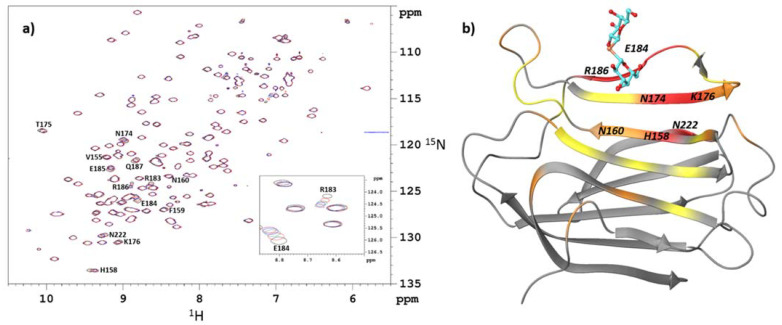
NMR titration of SeDG in the presence of *h*Gal-3. (**a**) Overlay of 500 MHz 2D ^15^N-^1^H HSQC spectra for 0.27 mM ^15^N-labeled *h*Gal-3 (CRD) obtained upon titration with SeDG. In the main map, cross peaks in black correspond to the reference HSQC spectrum recorded in the absence of SeDG; those in brown belong to the ^15^N-^1^H correlation map from the last titration step (0.4 mM of SeDG added). The inset shows the most shifted ^15^N/^1^H cross peaks (Arg183 and Glu184) as a function of increasing ligand concentrations (concentration of added ligand: 0.0, 0.1, 0.2, 0.3, and 0.4 mM, the corresponding cross peaks in black, red, green, blue, and brown, respectively). (**b**) Euclidean-weighted ^15^N- and ^1^H chemical shift changes [31] (Δ*d*) of *h*Gal-3 (CRD) determined between the end-points of titration with SeDG. Selected characteristic Δ*d* values are mapped on the X-ray structure of the *h*Gal-3 (CRD)-SeDG complex obtained in the present study. Amino acid residues of *h*Gal-3 (CRD) are color-coded as follows: Δ*d* > 0.02 red, Δ*d* = 0.01–0.02 orange, and Δ*d* = 0.006–0.01 yellow.

We recorded 2D ^15^N-^1^H HSQC spectra separately on samples of *U*-^15^N-enriched CRD of *h*Gal-3 in the presence of increasing amounts of either SeDG or DSeDG containing selenium in natural abundance [30]. An overlay of the spectra for the titration with SeDG is shown in Figure 1a with chemical shift changes plotted against the residue number depicted in Supplementary Figure S1. A shifting of some of the cross peaks on both the ^1^H and ^15^N chemical shift axis was observed in the presence of the ligand without appreciable line broadening. This indicates fast ligand exchange on the NMR time scale where the observed chemical shift is a weighted average of those in the free and bound states.

Not surprisingly, when mapping the most perturbed residues onto the structure of *h*Gal-3, we found that the chemical shifts of residues in S4 (154–165) and S5 (168–177) *β*-sheets near the inner (C) binding subsite are affected upon binding. Among them, His158 and Asn174 were mostly perturbed, accompanied with intermediate shifts of Thr175 and Lys176 in S5 *β*-strand, while Phe159 and Asn160 showed only minor changes in their Δ*d* (^15^N, ^1^H) values (Figure 1b).

In addition, all residues between 182 and 190 were affected with the Arg183, Glu184, Glu185, Arg186 and Gln187 cross peaks shifted most significantly. These data indicate contacts of SeDG with amino acid residues in the distal (*D* and *E*) binding subsite (S6 *β*-strand) as well. The solution structure of the *h*Gal-3 (CRD) complexed with SeDG should therefore be similar to that seen in the crystal structure or to the complex with TDG [32] occupying the canonical C/D binding region of the protein. It is to be noted, however, that “inner” and “distal” galactosyl residues (see Section 2.4) could not be distinguished in the NMR spectra because of molecular symmetry in the ligands.

For DSeDG, a similar pattern of cross peak displacements was observed in the 2D ^15^N-^1^H HSQC spectra of *h*Gal-3 (CRD), however, with markedly reduced chemical shift changes [Δ*d* (^15^N, ^1^H) (Figure S2)]. This is compatible with the crystallographic results discussed below and the *K_d_* values determined from ^15^N-^1^H HSQC titrations (below).

Chemical shift perturbation was, furthermore, observed in residues far from the carbohydrate binding site. Specifically, the Asn222 cross peak was perturbed most significantly (Figure 1 and Figure S1), which is located on the F-face of the CRD. Perturbations in further F-face signals may occur but reliable detection is hampered either by small values of binding-induced chemical shifts or an overlap in the ^15^N-^1^H HSQC spectra.

Several studies have been published [33,34,35,36,37,38,39] which map the carbohydrate recognition site of galectins via binding of small and high molecular weight carbohydrate ligands using 2D ^15^N-^1^H HSQC chemical shift titration as an established NMR technique. The involvement of residues in the F-face *β*-sheets, in addition to those in the canonical S-face binding site, have been reported in ^15^N HSQC binding studies of diLacNAc [34], CD146 glycan [37] or creatine sulphate [36] to *h*Gal-3. However, in the case of the chemical shift perturbation of Asn222 upon binding SeDG, the explanation is likely of a dynamic nature. Two different orientations were detected for the sidechain of Asn222 in the crystal structure of the SeDG–*h*Gal-3 complex (see Section 2.4); thus, the chemical shift perturbation might be an indication of a local dynamic process taking place between the two conformational states of this residue in the complex.

### 2.2. Determination of Binding Affinity by NMR Titrations

To assess the *K_d_* of *h*Gal-3 (CRD)*–*SeDG complex, nine residues showing the largest chemical shifts upon complex formation were selected (Figure S3a). The simultaneous fit of the binding curves yielded an equilibrium dissociation constant, *K_d_* = 123 ± 5 μM, indicating medium strength interaction between SeDG and *h*Gal-3 (CRD).

For DSeDG ^15^N HSQC, the analysis indicated significantly weaker interaction in the *h*Gal-3(CRD)*–*DSeDG complex. The individual fits of the binding curves (Figure S3b) for the two residues showing the highest sensitivity of chemical shifts upon titration, Thr175 and Asn174, yielded *K_d_*s of 11 mM and 6 mM, respectively. The relatively large uncertainty in the *K_d_* values is probably due to the inherent limitation of the chemical shift mapping approach in the weak binding regime. Notably, the reliable and accurate determination of *K_d_* from ^15^N-HSQC titration experiments is typically limited to the range of 10 mM > *K_d_* > 1 μM, thus, the current *K_d_* for DSeDG is close to the upper limit of applicability of the approach.

### 2.3. Determination of Binding Affinity by Fluorescence Anisotropy Titrations

**Figure 2:**
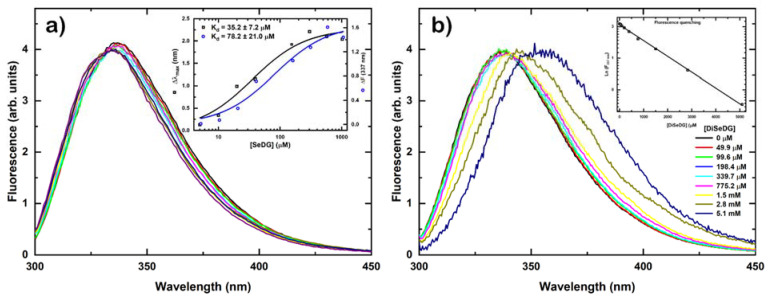
Fluorescence anisotropy measurements monitored by intrinsic Trp fluorescence at 25 °C. (**a**) Interaction of *h*Gal-3 with SeDG. The inset shows the binding isotherms for increasing concentrations of SeDG at 25 °C for the blue shift of the emission maximum wavelength (black squares) and for the fluorescence intensity at 337 nm (blue circles). Equilibrium *K_d_* values were derived by data fitting to a nonlinear regression model for single site-specific binding using GraphPad Prism 8 and assuming one binding site per monomer. *K_d_* errors are estimates from the fitting algorithm. (**b**) Interaction of *h*Gal-3 with DSeDG. The inset shows the inner filter effect produced by the addition of the ligand to the protein solution.

Residue W181 is known to engage in a CH-π stacking interaction with the hydrophobic β-face of the Gal ring and gives rise to an intrinsic fluorescence of the Trp residue. Fluorescence anisotropy measurements as shown in Figure 2a confirmed the presence of this specific stacking interaction for SeDG, however, no specific binding was detected for DSeDG below a ligand concentration of 0.8 mM (Figure 2b). Above this concentration a red shift of the relative emission maximum was observed, most likely not related to any specific binding, only due to the high ligand concentration. The inset shows the intrinsic fluorescence decrease produced by the addition of the ligand to the protein solution. This decrease follows a straight line indicating the lack of specific binding.

Furthermore, we also determined the strength of the interaction from the fluorescence anisotropy titrations and compared it to the values obtained by the NMR titrations and isothermal titration calorimetry (ITC) [40] as shown in Table 1. The dissociation constants found by NMR were 123 ± 5 μM for SeDG and 8500 ± 2500 μM for DSeDG, which were similar to the values by fluorescence anisotropy of 35.2 ± 7.2 for SeDG with no value available for DSeDG. These affinities were roughly in agreement with affinities of 93.1 ± 2.6 μM (SeDG) and 2800 ± 180 μM (DSeDG) [40] determined earlier by ITC measurements.

The binding strength of SeDG to *h*Gal-3 is somewhat smaller than that of the sulphur analogue TDG (67.6 ± 2.0 kcal/mol) [40], which in turn also binds somewhat less strongly than the endogenous ligand N-acetyllactosamine (38.6 ± 0.5 kcal/mol) [40]. Note that fluorescence anisotropy titration suggests no specific binding of DSeDG to *h*Gal-3, indicated by not applicable (n.a.) in Table 1.

### 2.4. Crystal Structures of SeDG and DSeDG in Complex with hGal-3 CRD

**Figure 3:**
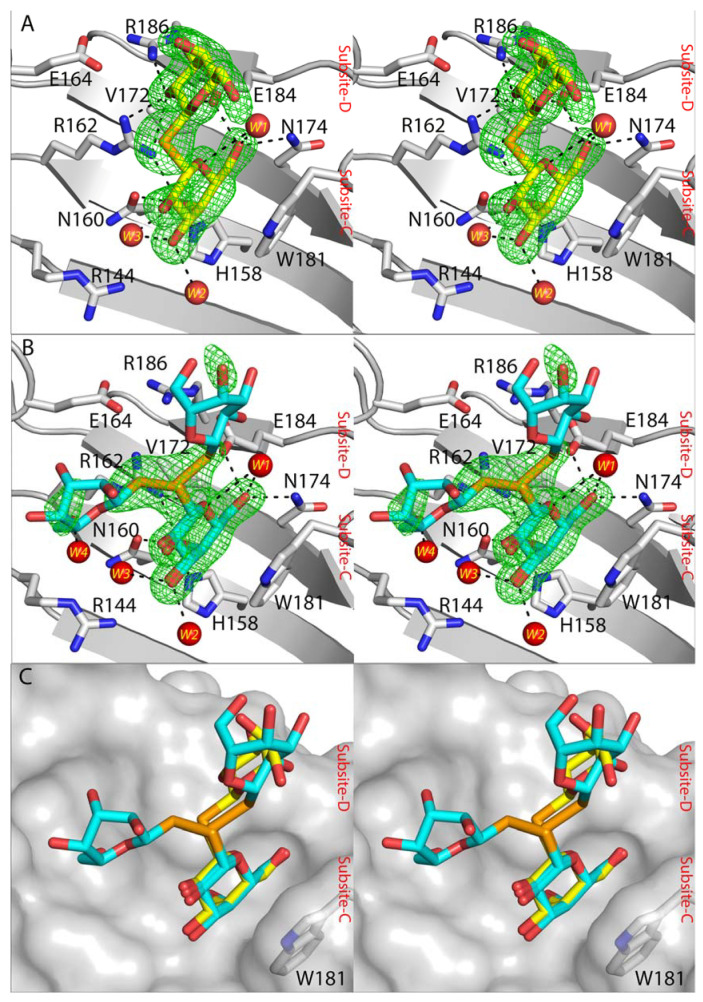
(**A**) 2│F_o_│ − │F_c_│ α_c_ map for SeDG (yellow colour sticks) (PDB ID 7RDP) in complex with *h*Gal-3 CRD at 1 σ. (**B**) DSeDG (cyan colour sticks) (PDB ID 7RDO) at 1 σ in complex with *h*Gal-3. (**C**) Alignment of SeDG and DSeDG molecules in complex with *h*Gal-3 CRD. *h*Gal-3 depicted as a grey ribbon and interacting residues indicated as grey sticks, hydrogen bonds indicated as a dashed black line. The water molecules interacting with both compounds are depicted as red spheres.

Crystal structures of *h*Gal-3 CRD with SeDG and DSeDG revealed that in both cases a galactose ring (here termed “inner” galactose ring) could bind within the carbohydrate binding site at subsite-C. A very strong peak within the CRD was revealed in the calculated anomalous difference map for the selenium atom in each of the SeDG and DSeDG structures. The Trp181 plays an important role in a stacking interaction with the inner galactose ring of both compounds. The inner galactose ring of both SeDG and DSeDG also exhibits several characteristic interactions with amino acid residues in the carbohydrate binding site. The C6 hydroxyl group (C6-OH) of the galactose ring participates in hydrogen bonding with the side chain of Asn174, Glu184 and a water molecule *W1* (Figure 3A). The oxygen atom (O5) of the galactose ring can interact with Arg162 and water molecule *W1*. The C4-OH participates in hydrogen bonding with His158, Asn160, Arg162 and the water molecule *W3*, whilst the C3-OH interacts with two water molecules (*W2* and *W3*). These interactions are consistent with those in the structures of galectin-3 CRD with bound galactose-based derivatives [41,42,43].

The second (“distal”) galactose ring of SeDG occupies subsite-D and forms fewer interactions with galectin-3 CRD. The C2′-OH of the distal galactose interacts with the side chain of Arg162, Glu184 and Arg186 of galectin-3 CRD, whilst the C3′-OH participates in hydrogen bonding with the side chain of Glu184. In contrast, the distal galactose ring of DSeDG and its Se–Se linkage show evidence of adopting two conformations (Figure 3B). One has ~30% occupancy for the distal galactose ring and has its Se–Se linkage orientated toward subsite-D, and the other (~70% occupancy) is oriented towards solvent. The electron density for DSeDG could not be unambiguously traced, however, based on the weak density associated with the distal galactose ring in the 70% occupancy conformation there is an indication that C6′-OH could have the ability to make a hydrogen bonding with water molecule *W4* (Figure 3B).

SeDG exhibits stronger binding affinity by ca. one order of magnitude to *h*Gal-3 CRD compared to DSeDG (see NMR and Section 3.5). The crystal structure of SeDG in complex with *h*Gal-3 CRD revealed that the Se-glycosidic bond caused a bending of the distal galactose ring toward subsite-D resulting in stable interactions between C2′-OH and the side chain of Arg162, Glu184 and Arg186 of *h*Gal-3 CRD. On the other hand, the Se–Se linkage of DSeDG is disordered and more extended within the solvent region. If the Se–Se linkage would orient the distal galactose ring towards subsite-D then there is potential for steric clashes with protein (Figure 3C). This offers an explanation for the lower affinity of the DSeDG compared to SeDG.

**Figure 4:**
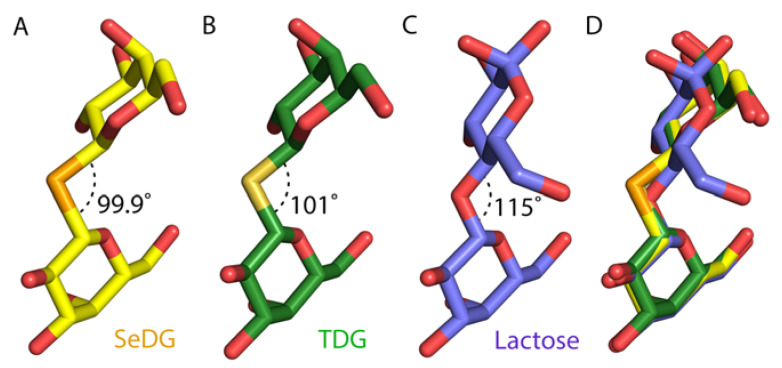
(**A**) Bond angle between C-Se-C in SeDG (yellow sticks) is 99.9° (PDB ID 7RDP). (**B**) Bond angle between C-S-C in thiodigalactoside (TDG, green sticks) is 101° (PDB ID 4JC1). (**C**) Bond angle between C-O-C in lactose (purple sticks) is 115° (PDB ID 2NN8). (**D**) Superimposition of SeDG, TDG and lactose indicates an identical position for inner galactose ring for all three molecules in the carbohydrate binding site of *h*Gal-3, whilst there is a slight deviation observed on SeDG distal galactose ring compared to glucose of lactose. However, the distal galactose of TDG and SeDG occupies an identical position within the *h*Gal-3 carbohydrate binding site.

A comparison of the atomic structures of *h*Gal-3 CRD SeDG complex with lactose [32] and TDG [32] indicates an identical position for the inner galactose ring for all three molecules (Figure 4D). The longer bond length in the selenium linkage in SeDG causes a slight shift of the distal galactose ring toward subsite-D compared to the glucose ring of lactose. However, the distal galactose of TDG and SeDG has an identical position in the subsite-D of galectin-3. The bond angle of C-Se-C (99.9°) is smaller than C-O-C in lactose (115°) and C-S-C (101°) in TDG (Figure 4A–C). Despite this difference in bond angle, C2′-OH of distal galactose ring of SeDG is still able to make hydrogen bonds with the Arg162, Glu184 and Arg186 side chains (Figure 4A). The combination of an increased C-Se bond length 1.4 Å to 1.9 Å and C-Se-C distance (2.4 Å to 3.0 Å) with a reduction of the bond angle from 115° to about 95° lets the O2 in TDG and SeDG become the equivalent of O3 in lactose.

### 2.5. Dynamics and Energetics of the Ligand Binding by Molecular Dynamics (MD) Simulations

Biomolecular simulations have become a method of choice to gain a detailed insight into the structure and dynamic properties of protein–ligand complexes [44,45,46,47,48]. To gain insight into the energetics of the selenium disaccharides binding to *h*Gal-3 CRD, we carried out multiple MD simulations for 1000 ns. For DSeDG, individual simulations were performed using the two bound ligand conformations. The binding mode with 70% occupancy is designated as binding mode A, while the one with 30% occupancy is named as binding mode B.

**Figure 5:**
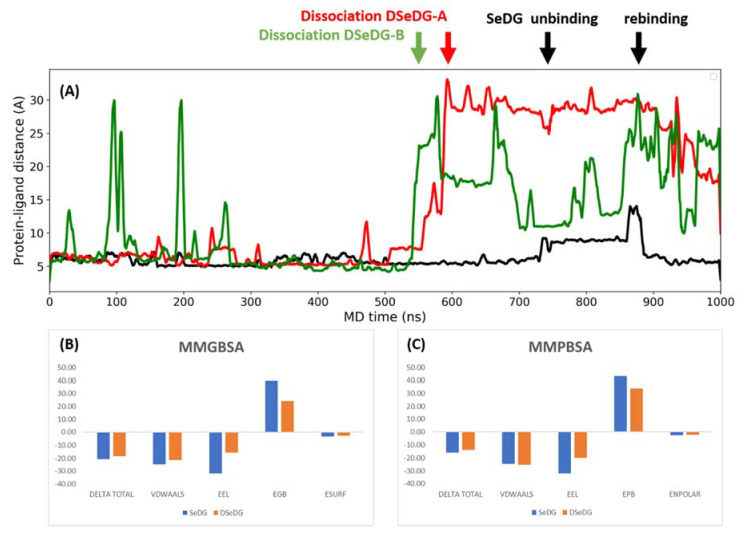
MD simulations started from the *h*Gal-3 CRD complexes. (**A**) Evolution of the distance between the subsite C and the ligand as measured by the distance between geometric centre of the Trp181 ring on the protein and the centre of the interglycosidic linkage of the ligands for SeDG (black), for DSeDG starting the simulation from binding mode A (red) and binding mode B (green). (**B**,**C**) The energetic contributions of the free energy of binding for SeDG in blue and DSeDG in orange to *h*Gal-3 CRD using the (**B**) MMGBSA and (**C**) MMPBSA calculations. The values only contain the binding enthalpy without the correction by entropic contributions to get the absolute values of free energies.

The average residence time of the ligand in the binding site was 730 ns for SeDG, while an average of 365 ns was observed for DSeDG. These residence times agree with the experimental dissociation constant of DSeDG being larger than that of SeDG. The dissociation event was tracked by the distance between the protein and the ligand as shown in representative simulations for the three complexes in Figure 5.

In this particular simulation, SeDG did not dissociate from *h*Gal-3 CRD during 1 μs simulation time, while in further replica simulations, dissociation did occur. Before full dissociation, multiple unbinding events were observed for DSeDG starting from binding mode B. There was no conversion between the two binding modes of DSeDG observed in the simulation time of 1 μs. In DSeDG, the dihedral around the diseleno bridge (C1-Se-Se′-C1′) started from +90.02° in binding mode A and is kept around this value in the simulation, while in binding mode B the starting dihedral is −125.53°, which averages to –90° during the simulation (Figure S4). Thus, binding mode A corresponds to a favourable arrangement of the interglycosidic dihedral of the ligand, but in mode B that is somewhat distorted from the ideal −90° by contacts with the protein.

To quantify the strength of the association between *h*Gal-3 CRD and the ligands as seen in the MD simulations, we performed MMGBSA and MMPBSA calculations [49] to obtain the free energy of the binding. The MMPB/GBSA calculations are end-state free energy methods calculating changes in energies over an ensemble of conformations obtained in simulations using implicit solvation models for estimating the solvation energy. The energy changes, however, only account for the binding enthalpy, while the entropic contribution needs to be accessed separately, but it is expected to be rather similar for analogous ligands.

The MMGBSA calculation showed a difference in the binding enthalpy between SeDG and DSeDG of 1.9 kcal/mol, while the MMPBSA yielded 2.1 kcal/mol difference, which corresponds to a ca. 50–500-fold larger dissociation constant for DSeDG than for SeDG. This trend is in good agreement with the affinities found experimentally, where ITC yielded approximately 30-times and the NMR titrations gave ca. 69-times larger affinity for SeDG over DSeDG (Table 1).

We also looked at the overall energetic contributions of the calculated free energies (Figure 5B,C). For the MMGB/PBSA calculation changes in the internal energy and in the solvation energy upon ligand binding are taken into consideration. The change in internal energy is composed of changes in the internal bonding terms, the van der Waals and the electrostatic terms, while the variation in the solvation energy is accounted for by an electrostatic and a nonpolar term. The electrostatic contribution to the solvation is calculated by the linear Poisson–Boltzmann equation for each conformer or by using simplified analytical methods of the generalized Born model. The nonpolar contribution to the solvation is accounted for by an attractive dispersion and repulsive cavity interaction. This term essentially represents the creation of a cavity in the solvent and intrinsically includes entropic effects related to solvation.

The breakdown of enthalpic contributions shows favourable changes in the van der Waals and electrostatic energies, which are due to new interactions formed between the protein and the ligand at the binding site. There is, however, a huge—but expected—unfavourable change in the electrostatic contribution of the solvation, which is associated with desolvation of the protein and the ligand upon forming the complex and aligning them at the binding interface; there is also a somewhat favourable change in the nonpolar part of the solvation as calculated by the attractive dispersion and repulsive cavity terms. While the changes in the van der Waals interactions and the nonpolar solvation terms are rather similar for SeDG and DSeDG, the changes in the internal electrostatic energies and electrostatics of the solvation energy upon ligand binding are more favourable for the SeDG than for DSeDG.

Decomposition of the energetic contribution on a per residue level (Table 2) showed that similar protein residues are contributing with favourable enthalpies to the binding of SeDG as to DSeDG. The enthalpic contribution of the ligand is larger for SeDG with similar favourable van der Waals and electrostatic contribution and a large unfavourable polar solvation energy with a negligible nonpolar part as detailed in Supplementary Table S2. The strongest protein contributor residue for both compounds is Trp181 dominated by a favourable van der Waals and electrostatic interaction and an unfavourable polar solvation energy. Contact residues are also among the larger contributors, such as Asn174 dominated by large negative electrostatics, some negative van der Waals interaction and a somewhat positive polar solvation energy; His158 with equally negative van der Waals and electrostatics, but with large positive polar solvation energy; Arg162 with large negative electrostatics, somewhat negative van der Waals and large positive polar solvation energy and finally Asn160 contributing predominantly with a favourable van der Waals interaction. The van der Waals and electrostatic interactions of these contact residues are likely attributable to the hydrogen bonds formed with the ligand. Hydrophobic residues with their sidechains in contact with the ligand also contribute, such Val172, Ala146, Val155 with a negative van der Waals energy and sometimes also with a favourable polar solvation energy. Interestingly, while their sidechain is not much in contact with the ligand, the backbones of Cys173 and Phe159 provide negative van der Waals energies towards the binding. Glu184 and Asp148 are interesting cases, as the residues have overall positive enthalpy changes which is the result of counterbalancing a favourable change in their van der Waals and electrostatic interactions with a large unfavourable polar solvation energy. Lys176 also has an overall small positive enthalpy change, however, this is a result of an unfavourable variation in its electrostatic energy upon ligand binding. All of these protein residues also show contributions to the binding in both simulations of DSeDG, but their magnitudes are generally smaller, which adds up to a smaller overall binding enthalpy. In addition to these main interactions, numerous smaller contributions are made by residues to DSeDG as shown in Supplementary Table S2, which are not present in SeDG.

## 3. Materials and Methods

### 3.1. Expression and Purification of Human Galectin-3 CRD

Human galectin-3 CRD (amino acid residues 108–250) was expressed and purified in its untagged form as described previously [32]. Briefly, two litres of bacterial culture were induced at OD600 of 0.6 with 1 mM IPTG and grown for 3–4 h at 37 °C. Bacterial cells were lysed, and galectin-3 CRD was purified through affinity chromatography on a lactosyl-sepharose column. Elution was performed using 100 mM lactose, and extensive dialysis was conducted in 1 × PBS to remove lactose. Finally, the protein was concentrated to 11.9 mg/mL and flash cryo-cooled in liquid nitrogen prior to storage at minus 80 °C.

### 3.2. Sample Preparation for NMR

For ^15^N-^1^H HSQC titration experiments, uniformly ^15^N-labeled human galectin-3 (CRD) was dissolved at a concentration of 0.270 mM in potassium phosphate buffer (10 mM) at pH 7.4, in 90% H_2_O/10% D_2_O solvent mixture, containing 0.5 M NaCl. The ^1^H and ^15^N resonance assignments for the carbohydrate recognition domain of human galectin-3 (*h*Gal-3 CRD) have already been reported [50] and used in the present study.

### 3.3. NMR Measurements

^15^N-^1^H HSQC NMR spectra were recorded on a Bruker Avance II spectrometer (Bruker BioSpin GmbH, Rheinstetten, Germany) operating at 500 MHz ^1^H frequency equipped with a TXI z-gradient probe. All experiments were performed at 303 K, and NMR data were processed with TopSpin 2.1 or 3.5 (Bruker Biospin GmbH, Karlsruhe, Germany) and analyzed with CcpNmr Analysis V2. Gradient- and sensitivity-enhanced 2D ^15^N-^1^H HSQC spectra were acquired with 128 *t*_1_ increments and 512 complex data points in *t*_2_ for digitizing nitrogen and proton frequency dimensions, respectively. To obtain adequate signal-to-noise in the resulting spectra, 64 scans were acquired for each *t*_1_ increment. Processing of raw data and analyzing (peak picking) of spectra were accomplished according to standard protocols implemented in TopSpin. For fitting of titration/binding curves, the minimization routine implemented in the Optimization Toolbox of Matlab R2015a was used. Error estimates of *K_d_* values were obtained by analyzing the quality of fit.

### 3.4. Determination of Dissociation Constants from NMR Titration

To quantitate binding-induced chemical shift changes, a measure *d* (average Euclidean distance), calculated from the ^15^N (*δ_N_*) and ^1^H (*δ_H_*) values, was used [31] (Equation (1)) with a scaling factor *α* = 0.14.

(1) $$d=\sqrt{\frac{1}{2}[\delta_{H}^{2}+{\left(\alpha \cdot \delta_{N}\right)}^{2}]}$$

*K_d_* (equilibrium dissociation constant) was then calculated by numerical fitting to the following equation:

(2) Δd=Δdmax{([P]t+[L]t+Kd)−[([P]t+[L]t+Kd)2−4[P]t[L]t]12} / 2[P]t

where Δ*d* is the change in the observed shift with reference to the free state and Δ*d*_max_ is the maximum of shift change obtained on saturation with ligand (fitted also), ${\left[P\right]}_{t}$ and ${\left[L\right]}_{t}$ are total protein and ligand concentrations, respectively.

### 3.5. Fluorescence Anisotropy

Fluorescence was measured using a Fluorolog-3 (Horiba Jobin Yvon) spectrofluorometer with excitation at 295 nm using 5 × 10 mm cells at 25 °C. Measurements were carried out at a protein concentration of 0.05 mg/mL in PBS (pH 7.0). The spectra were corrected for the buffer and ligand contribution, and for the dilution factor due to the addition of the ligand. The inner filter effect was corrected according to Mertens & Kagi [51]. Equilibrium *K_d_* values were determined by fitting the data to a nonlinear regression model for one site-specific binding using GraphPad Prism 8, assuming one binding site for *h*Gal-3. Figures were generated with ORIGIN2018 package (www.originlab.com, accessed on 11 January 2022).

### 3.6. Crystallization and Structure Determination

*h*Gal-3 CRD apo crystals were grown by the vapor-diffusion hanging-drop method with 500 μL reservoir solution (100 mM Tris-HCl, pH 7.0, 100 mM MgCl_2_, 31% *w*/*v* PEG 6000, 8 mM 2-mercaptoethanol) and 10 μL drops consisting of 5 μL protein solution (11.9 mg/mL galectin-3 CRD in PBS, pH 7.4) and 5 μL reservoir solution. Crystals appeared in 2–5 days and grew to a typical size of 0.1 × 0.1 × 0.5 mm in 1–2 weeks. The crystals were dipped in soaking solution (reservoir solution supplemented with 35 mM of SeDG and DSeDG) overnight (12–16 h). X-ray diffraction data sets were collected at 298 K at beamline MX1 of the Australian Synchrotron [52]. Data integration, scaling and merging were done using iMOSFLM and SCALA, in the CCP4 crystallographic software suite [53]. The atomic structures of the galectin-3 CRD SeDG and DSeDG complexes were solved by molecular replacement using a search model of the galectin-3 CRD (PDB ID: 2NMO [32]), and REFMAC5 was used for atomic model refinement [54]. Crystallographic data and model refinement statistics are given in Table S1. Visualization of electron densities and model building was performed using COOT [55]. Ligand geometry was obtained using the PRODRG2 server [56].

### 3.7. Molecular Dynamics

All MD simulations were performed using AMBER version 16 [57] implemented for GPUs [58,59]. The AMBER ff99SB [60] force field for the protein and the GAFF1 [61] force field for the carbohydrates with the TIP3P [62] model for water were used. Force field libraries for selenide and diselenide atom type were built with use of the R.E.D. server [63,64] using Gaussian 16 [65] as the QM engine. The cut-off used for nonbonded interactions was 8 Å. The particle-mesh Ewald [66] procedure is used to describe long-range electrostatic interactions with a maximal grid spacing of 1 Å. Periodic boundary conditions were applied using a truncated octahedron geometry. The SHAKE algorithm [67] was used to keep the bond lengths of hydrogen atoms rigid allowing a time step of 2 fs to be used. Protocol for NPT simulations: First, minimization in 2000 steps was performed, switching from steepest descent to conjugate gradient algorithm after 1000 steps. After minimization, a constant energy/constant volume (NVE ensemble) MD was carried out for 50 ps while increasing temperature from 0 K to 50 K. The system was further relaxed in a 1 ns long MD simulation using NPT ensemble while increasing temperature from 50 K to 310 K during 500 ps and keeping it at this temperature for 500 ps. During both relaxation steps backbone atoms were restrained with 4 kcal/molÅ^2^ force constant. Subsequently, a third relaxation step was conducted at 310 K and 1 bar pressure using an NPT ensemble for 1 ns without restraints. A 200 ns MD producing canonical NPT ensemble was carried out at 310 K and 1 bar. Both NTP ensembles were carried out using temperature regulation with Langevin dynamics with the collision frequency of 1 ps^−1^ and applying isotropic pressure scaling with pressure relaxation time of 1 ps. Analysis: The 50,000 coordinate snapshots were saved for analysis in all cases. The trajectories were analyzed with cpptraj [68] and visualized in VMD [69]. The binding-free energies were calculated by using the MMPBSA.py script [70] available in AmberTools using 1000 snapshots in the calculation of the binding enthalpy. Visualization and analysis of the molecules were done in PyMOL [71].

## 4. Conclusions

We have investigated the energetics, structure and dynamics of the interaction between selenoglycosides and *h*Gal-3 on the molecular level. Fluorescence anisotropy and ^15^N-^1^H HSQC NMR titrations provided confirmatory evidence for the canonical interaction pattern by C-H/π-interactions and H bonds in solution. For SeDG, fluorescence and NMR titrations gave a *K_d_*-value in the range of 35.2 ± 7.2 µM to 123.5 ± 5 µM, and titration calorimetry provided a value of 93.1 ± 2.6 µM [40]. Analysis of the cocrystal of *h*Gal-3 CRD complex with SeDG disclosed the canonical interaction pattern with galactose, as it has been described for *h*Gal-3 and Lac(NAc) in crystallography and NMR spectroscopy [13,72,73]. The distal pyranose of SeDG is able to contribute hydrogen bonding between its 2-OH and Arg162, Glu184 and Arg186. This stabilization of the overall contact fully resembles interactions of TDG with toad ovary and human Gal-1 [74,75] and the CRD of *h*Gal-3 [32]. The elongation of the distance between the two Gal-units in the DSeDG is not compatible with forming an ordered structure for the second galactose moiety, explaining its low-level bioactivity [40]. The free energy of binding calculations for MD simulations showed a major difference between the interaction of SeDG and DSeDG with *h*Gal-3 in the internal electrostatics as well as in the electrostatic part of the solvation. Breakdown of the binding enthalpy on a per residue level showed that overall, a similar set of residues are contributing to the binding of the two analogues, but the contributions for DSeDG are consistently smaller than for SeDG.

In summary, seleno analogues of natural ligands of *h*Gal-3 offer a unique, new set of compounds to become versatile tools in the interaction analysis, particularly by NMR spectroscopy, X crystallography or fluorescence analysis. Teaming up selenium labelling [30,76,77] with substitutions at strategic sites of a glycan by ^19^F [78,79] offers further intriguing possibilities, via different NMR time scales due to their individual chemical shift ranges, to analyze the structures and dynamics of the glycan ligand at a new level.

Furthermore, since bioisosteric substitution of the interglycosidic oxygen by selenium atom did not significantly reduce the binding strength of monoseleno-digalactoside (SeDG) compared to the endogenous ligand, but provides resistance against hydrolytic enzymes, the seleno analogue could serve as a novel scaffold for designing further *h*Gal-3 inhibitors. The design and synthesis of such compounds is underway in our laboratory.

## Figures and Tables

**Table 1:** Dissociation constants of selenoglycosides to *h*Gal-3 measured with different methods.

*K_d_* (µM)	SeDG	DSeDG
ITC [40]	93.1 ± 2.6	2800 ± 180
Fluorescence anisotropy titration	35.2 ± 7.2	n.a.
^15^N-^1^H HSQC titration	123 ± 5.0	8500 ± 2500

n.a.: no binding detected.

**Table 2:** Decomposition of enthalpic contributions of individual residues to the overall binding enthalpy sorted according to the values of SeDG.

MMGBSA					MMPBSA				
Residue		SeDG	DSeDG A	DSeDG B	Residue		SeDG	DSeDG A	DSeDG B
ligand		−10.98	−7.54	−8.84	ligand		−14.91	−11.84	−12.61
**TRP**	**181**	**−2.95**	**−1.60**	**−2.06**	**TRP**	**181**	**−2.87**	**−1.28**	**−1.70**
**ASN**	**174**	**−2.30**	**−0.77**	**−1.15**	**HIE**	**158**	**−1.14**	**−0.46**	**−0.24**
**HIE**	**158**	**−1.80**	**−0.83**	**−0.75**	**ASN**	**174**	**−1.07**	**−0.48**	**−0.54**
**ARG**	**162**	**−1.48**	**−0.42**	**−0.98**	**ASN**	**160**	**−1.01**	**−0.43**	**−0.63**
**ASN**	**160**	**−1.48**	**−0.66**	**−0.87**	**ARG**	**162**	**−0.92**	**−0.38**	**−0.72**
VAL	172	−1.04	−0.39	−0.52	VAL	172	−0.53	−0.25	−0.20
ALA	146	−0.71	−0.32	−0.27	ALA	146	−0.50	−0.24	−0.22
CYS	173	−0.61	−0.11	−0.22	ILE	145	−0.31	−0.10	−0.14
ARG	144	−0.56	−0.18	−0.31	CYS	173	−0.28	−0.05	−0.04
GLU	184	−0.36	−0.40	−0.12	PHE	159	−0.23	−0.07	−0.08
PHE	159	−0.36	−0.12	−0.14	GLU	165	−0.17	−0.04	−0.03
VAL	155	−0.28	−0.28	−0.08	PRO	161	−0.17	−0.06	−0.09
ILE	145	−0.26	−0.08	−0.12	LEU	147	−0.16	−0.06	−0.05
ARG	183	−0.21	−0.23	−0.06	VAL	155	−0.14	−0.20	−0.06
SER	237	−0.21	−0.03	−0.07	ASP	239	−0.13	−0.02	0.05
**LYS**	**176**	**0.14**	**0.00**	**0.07**	**LYS**	**176**	**0.16**	**0.06**	**−0.01**
**ARG**	**224**	**0.15**	**−0.19**	**0.10**	**ASP**	**148**	**0.87**	**0.58**	**0.51**
**ASP**	**148**	**0.16**	**−0.09**	**0.09**	**GLU**	**184**	**1.18**	**0.47**	**1.04**

## Data Availability

All data can be directly obtained by contacting the authors.

## Supplementary Materials

The following are available online at https://www.mdpi.com/article/10.3390/ijms23052494/s1.
