# Supplementary material for: Investigation of the Molecular Details of the Interactions of Selenoglycosides and Human Galectin-3
Source: Int J Mol Sci. 2022 Feb 24;23(5):2494. doi: 10.3390/ijms23052494 (PMC8910297; doi:10.3390/ijms23052494)
Supplement: Supplementary file 1 [file ijms-23-02494-s001.zip › ijms-1574529-supplementary.pdf]

Supporting Information

Article

Investigation of the Molecular Details of the Interactions of Selenoglycosides and Human Galectin-3

Mária Raics [1,†], Álex Kálmán Balogh [1,†], Chandan Kishor [2,†], István Timári [3], Francisco J. Medrano [4], Antonio Romero [4], Rob Marc Go [5], Helen Blanchard [5,*], László Szilágyi [3], Katalin E. Kövér [1,6,*] and Krisztina Fehér [1,*]

[1] Molecular Recognition and Interaction Research Group, Hungarian Academy of Sciences, University of Debrecen, Egyetem tér 1, H-4032 Debrecen, Hungary; raicsmaria@gmail.com (M.R.); balogh.alex@science.unideb.hu (Á.K.B.)
[2] School of Chemistry and Molecular Bioscience and Molecular Horizons, University of Wollongong, Wollongong, NSW 2522, Australia; chandan@uow.edu.au
[3] Department of Organic Chemistry, University of Debrecen, Egyetem tér 1, H-4032 Debrecen, Hungary; timari.istvan@science.unideb.hu (I.T.); lszilagyi@unideb.hu (L.S.)
[4] Structural and Chemical Biology, Centro de Investigaciones Biológicas, Margarita Salas, CSIC Ramiro de Maeztu 9, 28040 Madrid, Spain; fjmedrano@cib.csic.es (F.J.M.); romero@cib.csic.es (A.R.)
[5] Institute for Glycomics, Griffith University, Gold Coast, Queensland 4222, Australia; rob.go@alumni.griffithuni.edu.au
[6] Department of Inorganic and Analytical Chemistry, University of Debrecen, Egyetem tér 1, H-4032 Debrecen, Hungary
* Correspondence: h.blanchard@griffith.edu.au (H.B.); kover@science.unideb.hu (K.E.K.); feher.krisztina@science.unideb.hu (K.F.)
† These authors contributed equally.

Figure S1. Chemical shift mapping for SeDG by ^{15}N-^{1}H HSQC NMR titration.

Figure S2. ^{15}N-^{1}H HSQC NMR titration for DSeDG.

Figure S3. Determination of K_d values in the NMR titrations.

Table S1. Crystallographic data and refinement statistics.

Table S2. Energetic decomposition of binding enthalpy terms in MMGBSA and MMPBSA calculations.

Figure S4. The evolution of the C1-Se-Se-C1′ central dihedral angle in the DSeDG – hGal3 complex.

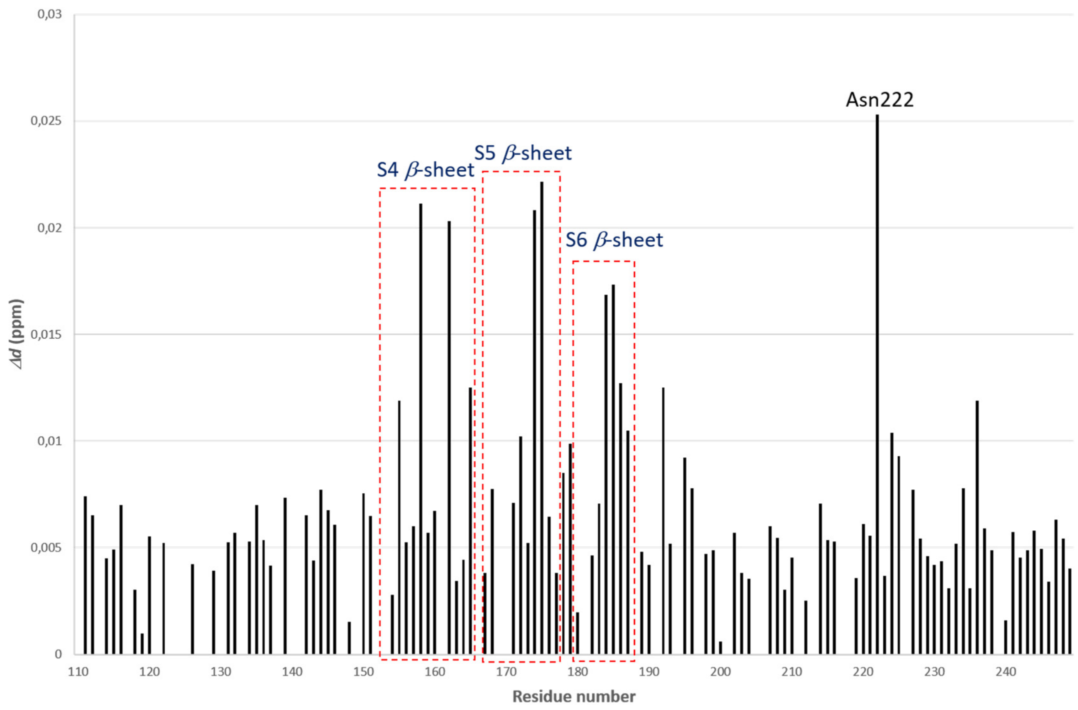

Figure S1. Euclidean weighted ^{15}N- and ^{1}H chemical shift changes (Δd) of *h*Gal-3 (CRD) determined between the end-points of titration with SeDG. Δd values are plotted as a function of residue number of *h*Gal-3 (CRD).

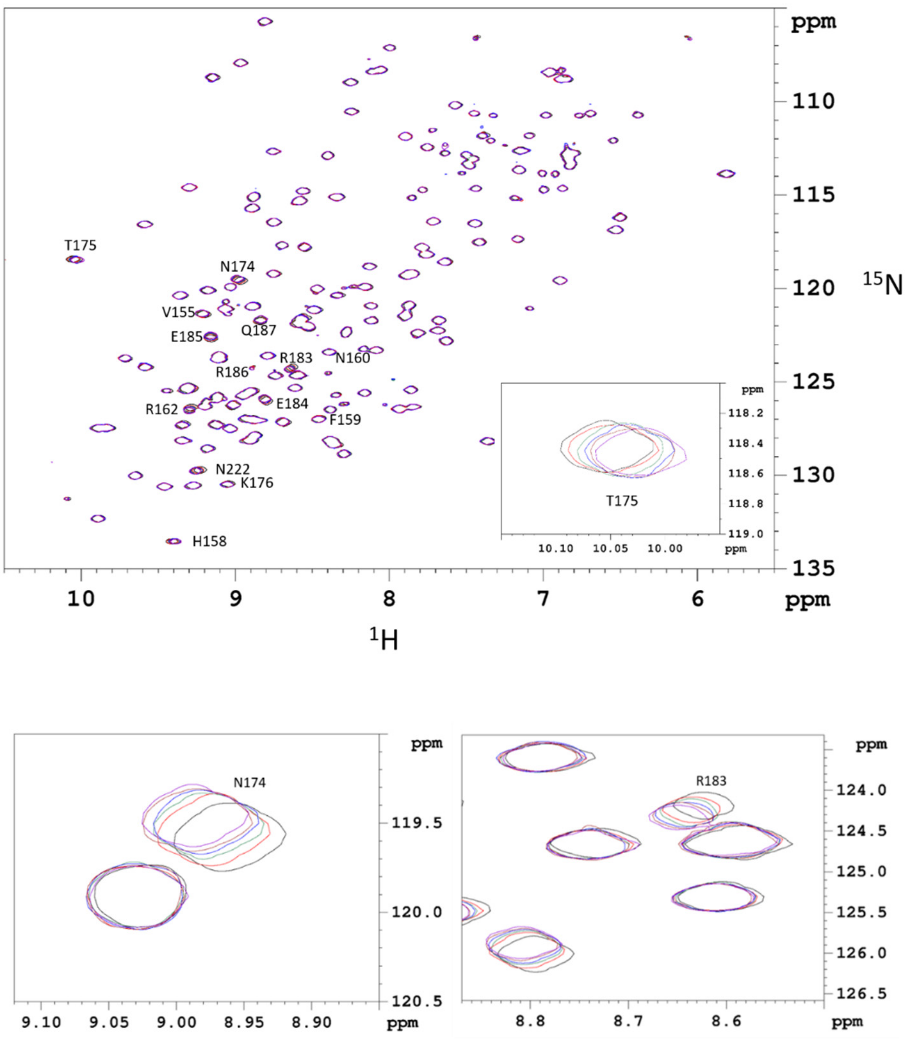

Figure S2. Overlay of ^{15}N-^{1}H HSQC spectra for 0.27 mM ^{15}N-labeled *h*Gal-3 (CRD) obtained upon titration with DSeDG (cc. of ligand: 0.0, 0.5, 1.0, 1.5, 2.0, and 2.5 mM). Cross peaks in black correspond to the reference HSQC-spectrum recorded in the absence of ligand DSeDG, while cross peaks in purple belong to the ^{15}N-^{1}H correlation map acquired in the last titration step. Cross peaks showing the largest shifts upon complex formation are assigned. The expansions of Thr175, Asn174 (subsite *C*) and Arg183 (subsite *D*) cross peaks with the most shifted ^{15}N- and ^{1}H resonances are shown in the inset and below the full spectrum, respectively.

(A)

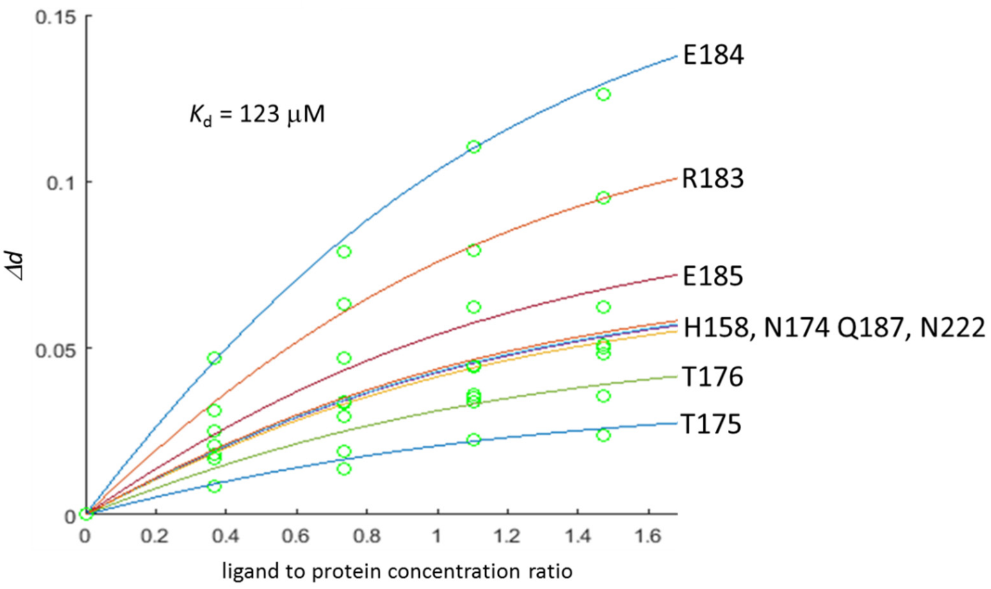

(B)

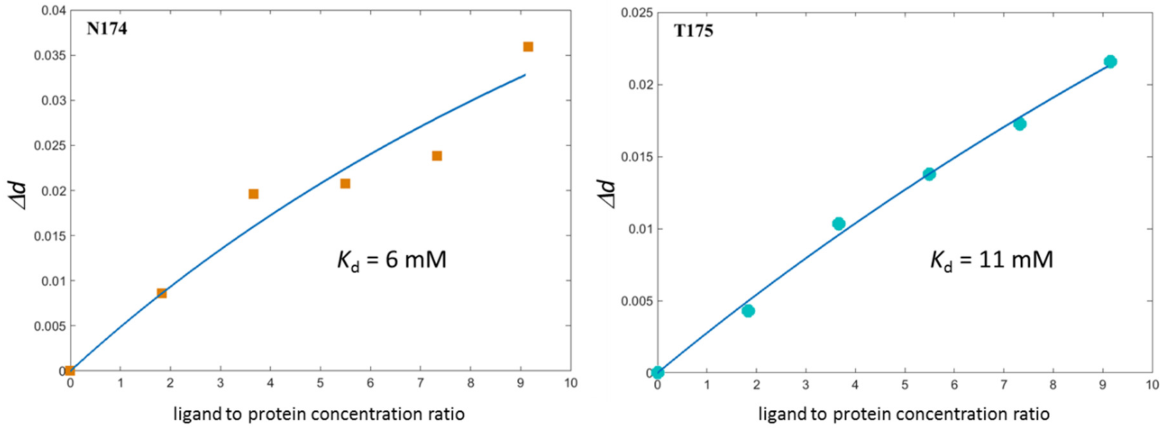

Figure S3. Evaluation of K_d based on ^{15}N,^{1}H chemical shift changes for *h*Gal-3 (CRD) induced by adding ligand *(A)* SeDG and *(B)* DSeDG. ^{15}N- and ^{1}H Euclidean weighted chemical shift changes (Δd) are plotted as a function of ligand to protein concentration ratio (A) for the 9 most affected residues and (B) the most shifted Asn174 and Thr175 residues. For fitting of binding curves the Optimization Toolbox of Matlab R2015a was used. During iterations the difference between the observed (Δd_{obs}) and calculated (Δd_{calc}) Δds was minimized with varying the values of K_d and Δd_{max}.

Table S1. Crystallographic data and refinement statistics of *h*Gal-3 CRD structures with bound SeDG and DSeDG.

<u>Parameters</u>	**hGal-3 CRD in complex with SeDG**	**hGal-3 CRD in complex with DSeDG**
Data collection		
Resolution range (Å)	30.84 - 1.96 (2.03 - 1.96)	31.53 - 2.0 (2.07 - 2.0)
Space group	$P2_12_12_1$	$P2_12_12_1$
Unit cell	*a* = 36.41, *b* = 58.03, *c* = 63.53	*a* = 34.90, *b* = 58.36, *c* = 63.05
Total reflections	39129	55226
Unique reflections	9282 (833)	9123 (884)
Multiplicity	4.2	6.0
Completeness (%)	91.46 (83.05)	99.5 (97.6)
Mean I/sigma(I)	20.54 (5.45)	11.0 (7.9)
Wilson B-factor	17.01	19.27
R-merge (%)	6.2 (13.9)	13.3 (20.0)
CC (1/2) [%]	99.9 (94.4)	98.4 (96.9)
Refinement Statistics		
R-work (%)	15.82 (18.10)	17.91 (15.59)
R-free (%)	18.72 (24.11)	23.09 (25.40)
RMS deviations		
RMS (bonds)	0.014	0.138
RMS (angles)	1.89	3.88
Ramachandran plot statistics (%)		
Favoured	98.54	97.81
Allowed	1.46	2.19
Outliers	0.00	0.00
PDB ID	7RDP	7RDO

Table S2. Energy decomposition of the binding enthalpy according to MMGBSA and MMPBSA calculations. Residues with large negative contributions are highlighted in red, residues with large positive contributions are shown in green.

a) SeDGal

	Res	VDW	Eel	PB	NPOL	TOTAL	VDW	Eel	PB	NPOL	TOTAL
SEG	112	-13.04	-15.21	19.56	-2.29	-10.98	-13.04	-15.21	13.34	0.00	-14.91
GLY	112	0.00	0.02	-0.01	0.00	0.01	0.00	0.02	-0.03	0.00	-0.01
PRO	113	0.00	-0.01	0.01	0.00	0.00	0.00	-0.01	0.01	0.00	0.00
LEU	114	0.00	0.00	0.00	0.00	0.01	0.00	0.00	0.00	0.00	0.00
ILE	115	0.00	-0.01	0.02	0.00	0.01	0.00	-0.01	0.01	0.00	0.00
VAL	116	-0.02	0.01	0.02	0.00	0.00	-0.02	0.01	0.00	0.00	-0.01
PRO	117	-0.05	0.03	0.01	-0.01	-0.02	-0.05	0.03	-0.02	0.00	-0.05
TYR	118	-0.01	-0.03	0.03	0.00	-0.01	-0.01	-0.03	0.03	0.00	0.00
ASN	119	-0.01	0.01	0.02	0.00	0.02	-0.01	0.01	-0.01	0.00	-0.01
LEU	120	0.00	0.00	0.01	0.00	0.00	0.00	0.00	0.01	0.00	0.00
PRO	121	0.00	0.00	0.00	0.00	0.00	0.00	0.00	0.00	0.00	0.00
LEU	122	0.00	0.00	0.01	0.00	0.00	0.00	0.00	0.00	0.00	0.00
PRO	123	0.00	0.00	0.00	0.00	0.00	0.00	0.00	0.00	0.00	0.00
GLY	124	0.00	0.00	0.00	0.00	0.00	0.00	0.00	0.00	0.00	0.00
GLY	125	0.00	0.00	0.00	0.00	0.01	0.00	0.00	0.00	0.00	0.00
VAL	126	0.00	0.00	0.01	0.00	0.01	0.00	0.00	0.00	0.00	0.00
VAL	127	0.00	0.00	0.00	0.00	0.00	0.00	0.00	0.00	0.00	0.00
PRO	128	0.00	0.00	0.00	0.00	0.00	0.00	0.00	0.01	0.00	0.00
ARG	129	0.00	-0.03	0.03	0.00	0.00	0.00	-0.03	0.02	0.00	-0.01
MET	130	0.00	0.01	0.00	0.00	0.00	0.00	0.01	-0.01	0.00	0.00
LEU	131	0.00	0.00	0.01	0.00	0.01	0.00	0.00	0.00	0.00	0.00
ILE	132	0.00	0.01	0.00	0.00	0.01	0.00	0.01	-0.01	0.00	0.00
THR	133	0.00	0.00	0.02	0.00	0.01	0.00	0.00	0.00	0.00	0.00
ILE	134	0.00	0.01	0.00	0.00	0.01	0.00	0.01	-0.01	0.00	0.00
LEU	135	0.00	0.00	0.01	0.00	0.00	0.00	0.00	0.00	0.00	0.00
GLY	136	0.00	0.00	0.01	0.00	0.01	0.00	0.00	0.00	0.00	0.00
THR	137	0.00	0.00	0.01	0.00	0.01	0.00	0.00	0.00	0.00	0.00
VAL	138	0.00	-0.01	0.04	0.00	0.02	0.00	-0.01	0.02	0.00	0.01
LYS	139	-0.01	-0.05	0.08	0.00	0.02	-0.01	-0.05	0.05	0.00	0.00
PRO	140	0.00	0.00	0.00	0.00	0.00	0.00	0.00	-0.01	0.00	0.00
ASN	141	0.00	0.00	0.01	0.00	0.01	0.00	0.00	0.00	0.00	0.00
ALA	142	-0.01	-0.01	0.04	0.00	0.03	-0.01	-0.01	0.03	0.00	0.01
ASN	143	-0.04	0.03	0.01	0.00	0.00	-0.04	0.03	-0.02	0.00	-0.03
ARG	144	-0.63	-1.12	1.32	-0.14	-0.56	-0.63	-1.12	1.80	0.00	0.05
ILE	145	-0.19	0.12	-0.19	0.00	-0.26	-0.19	0.12	-0.24	0.00	-0.31
ALA	146	-0.53	-0.08	-0.02	-0.08	-0.71	-0.53	-0.08	0.11	0.00	-0.50
LEU	147	-0.12	0.05	-0.03	0.00	-0.10	-0.12	0.05	-0.09	0.00	-0.16
ASP	148	-0.18	-1.15	1.52	-0.04	0.16	-0.18	-1.15	2.20	0.00	0.87
PHE	149	-0.02	-0.01	0.00	0.00	-0.03	-0.02	-0.01	0.01	0.00	-0.02
GLN	150	-0.03	0.06	-0.04	0.00	-0.01	-0.03	0.06	-0.09	0.00	-0.06
ARG	151	-0.01	0.03	0.01	0.00	0.03	-0.01	0.03	-0.03	0.00	0.00
GLY	152	0.00	-0.01	0.02	0.00	0.01	0.00	-0.01	0.01	0.00	0.00
ASN	153	-0.01	-0.03	0.06	0.00	0.02	-0.01	-0.03	0.03	0.00	-0.01

ASP	154	-0.01	-0.11	0.08	0.00	**-0.04**	-0.01	-0.11	0.10	0.00	**-0.02**
VAL	155	-0.14	0.02	-0.14	-0.01	**-0.28**	-0.14	0.02	-0.02	0.00	**-0.14**
ALA	156	-0.01	0.02	-0.01	0.00	**0.00**	-0.01	0.02	-0.03	0.00	**-0.01**
PHE	157	-0.05	-0.06	-0.05	0.00	**-0.17**	-0.05	-0.06	0.08	0.00	**-0.04**
HIE	158	-1.62	-1.54	1.54	-0.18	**-1.80**	-1.62	-1.54	2.02	0.00	**-1.14**
PHE	159	-0.18	-0.04	-0.14	0.00	**-0.36**	-0.18	-0.04	-0.02	0.00	**-0.23**
ASN	160	-1.27	0.11	-0.25	-0.07	**-1.48**	-1.27	0.11	0.15	0.00	**-1.01**
PRO	161	-0.07	0.09	0.08	0.00	**0.10**	-0.07	0.09	-0.19	0.00	**-0.17**
ARG	162	-1.10	-3.71	3.60	-0.26	**-1.48**	-1.10	-3.71	3.89	0.00	**-0.92**
PHE	163	-0.02	-0.02	0.04	0.00	**0.00**	-0.02	-0.02	0.04	0.00	**0.00**
ASN	164	-0.01	-0.03	0.08	0.00	**0.04**	-0.01	-0.03	0.04	0.00	**0.00**
GLU	165	-0.08	0.45	-0.43	0.00	**-0.06**	-0.08	0.45	-0.54	0.00	**-0.17**
ASN	166	-0.01	-0.01	0.03	0.00	**0.02**	-0.01	-0.01	0.01	0.00	**-0.01**
ASN	167	0.00	-0.01	0.03	0.00	**0.02**	0.00	-0.01	0.01	0.00	**0.00**
ARG	168	-0.01	-0.12	0.18	0.00	**0.05**	-0.01	-0.12	0.14	0.00	**0.01**
ARG	169	-0.01	-0.20	0.25	0.00	**0.05**	-0.01	-0.20	0.23	0.00	**0.02**
VAL	170	-0.02	0.04	-0.08	0.00	**-0.07**	-0.02	0.04	-0.06	0.00	**-0.05**
ILE	171	-0.04	-0.05	-0.09	0.00	**-0.18**	-0.04	-0.05	0.07	0.00	**-0.02**
VAL	172	-0.76	0.00	-0.25	-0.04	**-1.04**	-0.76	0.00	0.23	0.00	**-0.53**
CYS	173	-0.30	-0.11	-0.19	0.00	**-0.61**	-0.30	-0.11	0.13	0.00	**-0.28**
ASN	174	-0.70	-1.79	0.25	-0.06	**-2.30**	-0.70	-1.79	1.41	0.00	**-1.07**
THR	175	-0.10	0.09	-0.14	0.00	**-0.16**	-0.10	0.09	-0.09	0.00	**-0.10**
LYS	176	-0.18	0.37	-0.04	-0.01	**0.14**	-0.18	0.37	-0.02	0.00	**0.16**
LEU	177	-0.01	-0.01	0.03	0.00	**0.01**	-0.01	-0.01	0.00	0.00	**-0.02**
ASP	178	-0.01	-0.07	0.09	0.00	**0.02**	-0.01	-0.07	0.06	0.00	**-0.02**
ASN	179	-0.03	-0.03	0.11	0.00	**0.05**	-0.03	-0.03	0.04	0.00	**-0.02**
ASN	180	-0.07	0.02	0.03	0.00	**-0.02**	-0.07	0.02	0.02	0.00	**-0.03**
TRP	181	-3.03	-0.70	1.16	-0.38	**-2.95**	-3.03	-0.70	0.86	0.00	**-2.87**
GLY	182	-0.15	-0.11	0.25	-0.01	**-0.03**	-0.15	-0.11	0.22	0.00	**-0.05**
ARG	183	-0.15	0.03	-0.08	-0.01	**-0.21**	-0.15	0.03	0.02	0.00	**-0.10**
GLU	184	-0.15	-5.98	5.89	-0.12	**-0.36**	-0.15	-5.98	7.31	0.00	**1.18**
GLU	185	-0.06	0.24	-0.21	0.00	**-0.02**	-0.06	0.24	-0.31	0.00	**-0.12**
ARG	186	-0.15	0.14	0.01	-0.01	**-0.01**	-0.15	0.14	-0.05	0.00	**-0.05**
GLN	187	-0.01	0.01	0.01	0.00	**0.01**	-0.01	0.01	-0.02	0.00	**-0.02**
SER	188	0.00	0.01	0.00	0.00	**0.00**	0.00	0.01	-0.01	0.00	**0.00**
VAL	189	0.00	0.02	-0.01	0.00	**0.01**	0.00	0.02	-0.03	0.00	**-0.01**
PHE	190	-0.01	-0.01	0.00	0.00	**-0.02**	-0.01	-0.01	0.02	0.00	**0.00**
PRO	191	0.00	-0.01	0.01	0.00	**0.00**	0.00	-0.01	0.01	0.00	**0.00**
PHE	192	-0.01	0.00	-0.02	0.00	**-0.02**	-0.01	0.00	0.00	0.00	**0.00**
GLU	193	0.00	0.14	-0.13	0.00	**0.01**	0.00	0.14	-0.14	0.00	**0.00**
SER	194	0.00	0.00	0.00	0.00	**0.00**	0.00	0.00	0.00	0.00	**0.00**
GLY	195	0.00	-0.01	0.01	0.00	**0.00**	0.00	-0.01	0.01	0.00	**0.00**
LYS	196	0.00	-0.08	0.08	0.00	**0.00**	0.00	-0.08	0.06	0.00	**-0.02**
PRO	197	0.00	0.01	0.00	0.00	**0.00**	0.00	0.01	-0.01	0.00	**0.00**
PHE	198	0.00	0.00	-0.01	0.00	**-0.02**	0.00	0.00	0.00	0.00	**0.00**
LYS	199	0.00	-0.06	0.07	0.00	**0.02**	0.00	-0.06	0.03	0.00	**-0.02**
ILE	200	0.00	-0.01	0.02	0.00	**0.01**	0.00	-0.01	0.01	0.00	**0.00**
GLN	201	0.00	0.01	0.00	0.00	**0.01**	0.00	0.01	-0.01	0.00	**0.00**
VAL	202	0.00	-0.01	0.02	0.00	**0.01**	0.00	-0.01	0.01	0.00	**0.00**
LEU	203	0.00	0.01	0.00	0.00	**0.00**	0.00	0.01	-0.01	0.00	**0.00**
VAL	204	0.00	0.00	0.01	0.00	**0.01**	0.00	0.00	0.00	0.00	**0.00**

GLU	205	0.00	0.06	-0.06	0.00	**0.00**	0.00	0.06	-0.05	0.00	**0.01**
PRO	206	0.00	0.00	0.00	0.00	**0.00**	0.00	0.00	0.00	0.00	**0.00**
ASP	207	0.00	0.06	-0.06	0.00	**0.00**	0.00	0.06	-0.06	0.00	**0.00**
HIE	208	0.00	0.00	0.02	0.00	**0.01**	0.00	0.00	0.00	0.00	**0.00**
PHE	209	0.00	0.01	-0.02	0.00	**-0.02**	0.00	0.01	-0.01	0.00	**-0.01**
LYS	210	0.00	-0.09	0.10	0.00	**0.01**	0.00	-0.09	0.08	0.00	**-0.01**
VAL	211	0.00	0.00	0.02	0.00	**0.01**	0.00	0.00	0.00	0.00	**0.00**
ALA	212	0.00	0.00	0.00	0.00	**0.00**	0.00	0.00	0.00	0.00	**0.00**
VAL	213	0.00	0.00	0.02	0.00	**0.01**	0.00	0.00	0.00	0.00	**0.00**
ASN	214	0.00	0.00	0.00	0.00	**0.00**	0.00	0.00	0.00	0.00	**0.00**
ASP	215	0.00	0.06	-0.06	0.00	**0.00**	0.00	0.06	-0.04	0.00	**0.02**
ALA	216	0.00	0.01	0.00	0.00	**0.00**	0.00	0.01	-0.01	0.00	**0.00**
HIE	217	0.00	0.00	0.01	0.00	**0.01**	0.00	0.00	0.00	0.00	**0.00**
LEU	218	0.00	0.00	0.03	0.00	**0.02**	0.00	0.00	0.00	0.00	**0.00**
LEU	219	-0.01	-0.01	0.02	0.00	**0.00**	-0.01	-0.01	0.01	0.00	**0.00**
GLN	220	0.00	0.01	0.02	0.00	**0.02**	0.00	0.01	-0.01	0.00	**0.00**
TYR	221	-0.03	0.02	-0.04	0.00	**-0.05**	-0.03	0.02	-0.02	0.00	**-0.02**
ASN	222	0.00	0.02	-0.01	0.00	**0.01**	0.00	0.02	-0.02	0.00	**0.00**
HIE	223	0.00	0.00	0.01	0.00	**0.01**	0.00	0.00	-0.01	0.00	**0.00**
ARG	224	-0.02	-0.35	0.52	0.00	**0.15**	-0.02	-0.35	0.35	0.00	**-0.02**
VAL	225	0.00	0.00	0.01	0.00	**0.01**	0.00	0.00	0.00	0.00	**0.00**
LYS	226	0.00	-0.04	0.05	0.00	**0.00**	0.00	-0.04	0.04	0.00	**0.00**
LYS	227	0.00	0.00	0.00	0.00	**0.01**	0.00	0.00	-0.01	0.00	**-0.01**
LEU	228	0.00	0.00	0.01	0.00	**0.00**	0.00	0.00	0.00	0.00	**0.00**
ASN	229	0.00	0.00	0.01	0.00	**0.00**	0.00	0.00	0.00	0.00	**0.00**
GLU	230	0.00	-0.03	0.04	0.00	**0.01**	0.00	-0.03	0.04	0.00	**0.00**
ILE	231	0.00	0.00	0.01	0.00	**0.01**	0.00	0.00	0.00	0.00	**0.00**
SER	232	0.00	0.00	0.00	0.00	**0.00**	0.00	0.00	0.00	0.00	**0.00**
LYS	233	-0.01	0.18	-0.14	0.00	**0.04**	-0.01	0.18	-0.19	0.00	**-0.02**
LEU	234	-0.01	0.00	-0.03	0.00	**-0.04**	-0.01	0.00	-0.02	0.00	**-0.02**
GLY	235	-0.02	0.05	-0.05	0.00	**-0.02**	-0.02	0.05	-0.03	0.00	**0.00**
ILE	236	-0.03	-0.02	0.00	0.00	**-0.06**	-0.03	-0.02	0.04	0.00	**-0.02**
SER	237	-0.29	-0.05	0.18	-0.05	**-0.21**	-0.29	-0.05	0.22	0.00	**-0.11**
GLY	238	-0.13	-0.10	0.14	-0.02	**-0.10**	-0.13	-0.10	0.16	0.00	**-0.07**
ASP	239	-0.05	0.18	-0.17	0.00	**-0.04**	-0.05	0.18	-0.27	0.00	**-0.13**
ILE	240	-0.02	0.00	0.02	0.00	**0.00**	-0.02	0.00	0.01	0.00	**-0.02**
ASP	241	0.00	0.04	-0.04	0.00	**0.00**	0.00	0.04	-0.04	0.00	**-0.01**
LEU	242	0.00	0.00	0.01	0.00	**0.01**	0.00	0.00	0.00	0.00	**0.00**
THR	243	0.00	0.00	0.01	0.00	**0.00**	0.00	0.00	0.00	0.00	**0.00**
SER	244	0.00	0.00	0.00	0.00	**0.00**	0.00	0.00	-0.01	0.00	**0.00**
ALA	245	0.00	0.00	0.01	0.00	**0.01**	0.00	0.00	0.00	0.00	**0.00**
SER	246	0.00	0.00	0.00	0.00	**0.00**	0.00	0.00	0.00	0.00	**0.00**
TYR	247	0.00	0.00	0.00	0.00	**0.00**	0.00	0.00	0.00	0.00	**0.00**
THR	248	0.00	0.00	0.00	0.00	**0.00**	0.00	0.00	0.00	0.00	**0.00**
MET	249	0.00	0.00	0.00	0.00	**0.00**	0.00	0.00	0.00	0.00	**0.00**
ILE	250	0.00	0.02	-0.02	0.00	**0.00**	0.00	0.02	-0.01	0.00	**0.01**

b) DSeDGal A binding mode

	Res.	VDW	Eel	PB	NPOL	TOTAL	VDW	Eel	PB	NPOL	TOTAL
SES	112	-10.45	-13.36	18.19	-1.93	-7.54	-10.45	-13.36	11.97	0.00	-11.84

GLY	112	0.00	0.00	0.01	0.00	0.00	0.00	0.00	0.00	0.00	-0.01
PRO	113	0.00	0.00	0.00	0.00	0.00	0.00	0.00	0.00	0.00	0.00
LEU	114	0.00	0.00	0.00	0.00	0.00	0.00	0.00	0.00	0.00	0.00
ILE	115	0.00	-0.01	0.01	0.00	0.00	0.00	-0.01	0.01	0.00	0.00
VAL	116	-0.01	0.01	0.01	0.00	0.01	-0.01	0.01	-0.01	0.00	-0.01
PRO	117	-0.02	0.01	0.00	0.00	-0.02	-0.02	0.01	-0.01	0.00	-0.03
TYR	118	-0.01	-0.03	0.03	0.00	-0.01	-0.01	-0.03	0.03	0.00	-0.01
ASN	119	-0.03	-0.02	0.04	0.00	-0.01	-0.03	-0.02	0.02	0.00	-0.02
LEU	120	0.00	0.00	0.01	0.00	0.00	0.00	0.00	0.01	0.00	0.00
PRO	121	-0.01	0.00	0.00	0.00	0.00	-0.01	0.00	0.00	0.00	-0.01
LEU	122	-0.01	-0.01	0.01	0.00	0.00	-0.01	-0.01	0.01	0.00	-0.01
PRO	123	-0.02	-0.01	0.01	0.00	-0.02	-0.02	-0.01	0.01	0.00	-0.01
GLY	124	-0.02	-0.02	0.03	-0.01	-0.02	-0.02	-0.02	0.03	0.00	-0.01
GLY	125	-0.02	0.00	0.01	0.00	0.00	-0.02	0.00	0.00	0.00	-0.02
VAL	126	-0.02	0.01	-0.01	0.00	-0.01	-0.02	0.01	0.00	0.00	0.00
VAL	127	-0.04	-0.02	0.01	-0.01	-0.06	-0.04	-0.02	0.02	0.00	-0.04
PRO	128	-0.08	-0.02	0.04	-0.01	-0.07	-0.08	-0.02	0.02	0.00	-0.08
ARG	129	-0.01	-0.04	0.04	0.00	0.00	-0.01	-0.04	0.03	0.00	-0.01
MET	130	0.00	0.00	0.00	0.00	0.00	0.00	0.00	0.00	0.00	0.00
LEU	131	0.00	0.00	0.01	0.00	0.01	0.00	0.00	0.00	0.00	0.00
ILE	132	0.00	0.01	0.00	0.00	0.00	0.00	0.01	-0.01	0.00	0.00
THR	133	0.00	0.00	0.01	0.00	0.01	0.00	0.00	0.00	0.00	0.00
ILE	134	0.00	0.00	0.00	0.00	0.00	0.00	0.00	-0.01	0.00	0.00
LEU	135	0.00	0.00	0.00	0.00	0.00	0.00	0.00	0.00	0.00	0.00
GLY	136	0.00	0.00	0.00	0.00	0.00	0.00	0.00	0.00	0.00	0.00
THR	137	0.00	0.00	0.01	0.00	0.00	0.00	0.00	0.00	0.00	0.00
VAL	138	0.00	-0.01	0.02	0.00	0.01	0.00	-0.01	0.01	0.00	0.00
LYS	139	-0.01	-0.05	0.07	0.00	0.01	-0.01	-0.05	0.05	0.00	-0.01
PRO	140	-0.01	0.00	0.00	0.00	0.00	-0.01	0.00	0.00	0.00	0.00
ASN	141	-0.03	-0.01	0.03	-0.01	-0.01	-0.03	-0.01	0.03	0.00	0.00
ALA	142	-0.01	-0.04	0.05	0.00	0.00	-0.01	-0.04	0.03	0.00	-0.01
ASN	143	-0.05	0.01	0.02	-0.01	-0.02	-0.05	0.01	0.00	0.00	-0.04
ARG	144	-0.28	-0.58	0.74	-0.06	-0.18	-0.28	-0.58	0.84	0.00	-0.02
ILE	145	-0.07	0.10	-0.11	0.00	-0.08	-0.07	0.10	-0.13	0.00	-0.10
ALA	146	-0.23	-0.07	0.01	-0.04	-0.32	-0.23	-0.07	0.06	0.00	-0.24
LEU	147	-0.10	0.08	-0.04	0.00	-0.06	-0.10	0.08	-0.05	0.00	-0.06
ASP	148	-0.03	-2.15	2.12	-0.03	-0.09	-0.03	-2.15	2.77	0.00	0.58
PHE	149	-0.02	-0.01	0.00	0.00	-0.04	-0.02	-0.01	0.01	0.00	-0.02
GLN	150	-0.12	0.05	-0.01	-0.02	-0.10	-0.12	0.05	-0.04	0.00	-0.12
ARG	151	-0.07	-0.06	0.15	-0.01	0.01	-0.07	-0.06	0.07	0.00	-0.07
GLY	152	-0.04	-0.04	0.07	-0.01	-0.03	-0.04	-0.04	0.07	0.00	-0.02
ASN	153	-0.09	-0.25	0.26	-0.03	-0.11	-0.09	-0.25	0.27	0.00	-0.07
ASP	154	-0.09	-0.24	0.23	0.00	-0.12	-0.09	-0.24	0.38	0.00	0.04
VAL	155	-0.22	0.02	-0.04	-0.03	-0.28	-0.22	0.02	0.01	0.00	-0.20
ALA	156	-0.02	0.03	0.00	0.00	0.02	-0.02	0.03	-0.04	0.00	-0.03
PHE	157	-0.03	-0.05	0.02	0.00	-0.06	-0.03	-0.05	0.07	0.00	-0.01
HIE	158	-0.82	-0.60	0.68	-0.08	-0.83	-0.82	-0.60	0.96	0.00	-0.46
PHE	159	-0.07	-0.01	-0.03	0.00	-0.12	-0.07	-0.01	0.02	0.00	-0.07
ASN	160	-0.58	0.18	-0.23	-0.04	-0.66	-0.58	0.18	-0.03	0.00	-0.43
PRO	161	-0.03	0.05	0.02	0.00	0.03	-0.03	0.05	-0.08	0.00	-0.06
ARG	162	-0.56	-1.92	2.19	-0.12	-0.42	-0.56	-1.92	2.10	0.00	-0.38

PHE	163	-0.02	-0.03	0.03	0.00	-0.02	-0.02	-0.03	0.03	0.00	-0.02
ASN	164	-0.06	-0.07	0.10	-0.01	-0.03	-0.06	-0.07	0.10	0.00	-0.02
GLU	165	-0.10	-0.05	0.03	-0.01	-0.13	-0.10	-0.05	0.12	0.00	-0.04
ASN	166	-0.09	-0.07	0.13	-0.02	-0.05	-0.09	-0.07	0.13	0.00	-0.03
ASN	167	-0.05	-0.06	0.09	-0.01	-0.02	-0.05	-0.06	0.07	0.00	-0.03
ARG	168	-0.17	-0.20	0.31	-0.03	-0.10	-0.17	-0.20	0.29	0.00	-0.09
ARG	169	-0.06	-0.23	0.27	-0.01	-0.02	-0.06	-0.23	0.26	0.00	-0.03
VAL	170	-0.11	-0.01	-0.02	-0.01	-0.14	-0.11	-0.01	0.00	0.00	-0.11
ILE	171	-0.03	-0.01	-0.01	0.00	-0.06	-0.03	-0.01	0.02	0.00	-0.03
VAL	172	-0.30	-0.02	-0.06	-0.02	-0.39	-0.30	-0.02	0.07	0.00	-0.25
CYS	173	-0.08	-0.01	-0.02	0.00	-0.11	-0.08	-0.01	0.04	0.00	-0.05
ASN	174	-0.33	-0.68	0.28	-0.03	-0.77	-0.33	-0.68	0.53	0.00	-0.48
THR	175	-0.15	0.00	0.01	-0.01	-0.14	-0.15	0.00	0.00	0.00	-0.14
LYS	176	-0.26	-0.40	0.69	-0.03	0.00	-0.26	-0.40	0.72	0.00	0.06
LEU	177	-0.26	-0.13	0.14	-0.03	-0.27	-0.26	-0.13	0.18	0.00	-0.20
ASP	178	-0.06	-0.58	0.66	-0.02	0.00	-0.06	-0.58	0.79	0.00	0.14
ASN	179	-0.08	-0.01	0.09	-0.01	-0.01	-0.08	-0.01	0.06	0.00	-0.03
ASN	180	-0.13	-0.26	0.21	-0.02	-0.20	-0.13	-0.26	0.19	0.00	-0.20
TRP	181	-1.54	-0.33	0.48	-0.21	-1.60	-1.54	-0.33	0.59	0.00	-1.28
GLY	182	-0.21	-0.21	0.25	-0.03	-0.20	-0.21	-0.21	0.33	0.00	-0.09
ARG	183	-0.28	-0.23	0.32	-0.04	-0.23	-0.28	-0.23	0.34	0.00	-0.16
GLU	184	-0.05	-2.56	2.27	-0.05	-0.40	-0.05	-2.56	3.08	0.00	0.47
GLU	185	-0.05	0.14	-0.09	0.00	0.01	-0.05	0.14	-0.11	0.00	-0.02
ARG	186	-0.27	-0.35	0.47	-0.04	-0.19	-0.27	-0.35	0.45	0.00	-0.17
GLN	187	-0.12	-0.02	0.03	-0.01	-0.12	-0.12	-0.02	0.06	0.00	-0.08
SER	188	-0.12	-0.10	0.15	-0.03	-0.10	-0.12	-0.10	0.16	0.00	-0.07
VAL	189	-0.02	0.02	-0.02	0.00	-0.01	-0.02	0.02	-0.03	0.00	-0.02
PHE	190	-0.01	-0.01	0.01	0.00	-0.02	-0.01	-0.01	0.02	0.00	-0.01
PRO	191	0.00	-0.01	0.01	0.00	0.00	0.00	-0.01	0.01	0.00	0.00
PHE	192	0.00	0.00	-0.01	0.00	-0.01	0.00	0.00	0.00	0.00	0.00
GLU	193	0.00	0.11	-0.10	0.00	0.01	0.00	0.11	-0.10	0.00	0.01
SER	194	-0.01	-0.01	0.01	0.00	0.00	-0.01	-0.01	0.01	0.00	0.00
GLY	195	0.00	0.00	0.01	0.00	0.00	0.00	0.00	0.01	0.00	0.00
LYS	196	0.00	-0.07	0.07	0.00	0.00	0.00	-0.07	0.06	0.00	-0.01
PRO	197	0.00	0.01	0.00	0.00	0.00	0.00	0.01	-0.01	0.00	0.00
PHE	198	0.00	0.00	0.00	0.00	-0.01	0.00	0.00	0.00	0.00	0.00
LYS	199	0.00	-0.04	0.05	0.00	0.01	0.00	-0.04	0.03	0.00	-0.01
ILE	200	0.00	-0.01	0.01	0.00	0.00	0.00	-0.01	0.01	0.00	0.00
GLN	201	0.00	0.01	0.00	0.00	0.01	0.00	0.01	-0.01	0.00	0.00
VAL	202	0.00	0.00	0.01	0.00	0.01	0.00	0.00	0.00	0.00	0.00
LEU	203	0.00	0.00	0.00	0.00	0.00	0.00	0.00	0.00	0.00	0.00
VAL	204	-0.01	0.00	0.01	0.00	0.00	-0.01	0.00	0.00	0.00	-0.01
GLU	205	-0.01	0.06	-0.06	0.00	-0.01	-0.01	0.06	-0.04	0.00	0.01
PRO	206	-0.07	-0.04	0.07	-0.01	-0.05	-0.07	-0.04	0.10	0.00	-0.02
ASP	207	-0.03	-0.05	0.09	0.00	0.01	-0.03	-0.05	0.12	0.00	0.04
HIE	208	-0.01	-0.01	0.02	0.00	0.00	-0.01	-0.01	0.01	0.00	-0.01
PHE	209	-0.01	0.00	-0.02	0.00	-0.02	-0.01	0.00	0.00	0.00	-0.01
LYS	210	0.00	-0.07	0.08	0.00	0.01	0.00	-0.07	0.05	0.00	-0.01
VAL	211	0.00	0.00	0.01	0.00	0.01	0.00	0.00	0.00	0.00	0.00
ALA	212	0.00	0.00	0.00	0.00	0.00	0.00	0.00	0.00	0.00	0.00
VAL	213	0.00	0.00	0.01	0.00	0.01	0.00	0.00	0.00	0.00	0.00

ASN	214	0.00	0.00	0.00	0.00	0.00	0.00	0.00	0.00	0.00	0.00
ASP	215	0.00	0.05	-0.05	0.00	0.00	0.00	0.05	-0.04	0.00	0.01
ALA	216	0.00	0.00	0.00	0.00	0.00	0.00	0.00	0.00	0.00	0.00
HIE	217	0.00	0.00	0.01	0.00	0.01	0.00	0.00	0.00	0.00	0.00
LEU	218	0.00	0.00	0.02	0.00	0.01	0.00	0.00	0.00	0.00	0.00
LEU	219	-0.01	-0.01	0.02	0.00	0.00	-0.01	-0.01	0.01	0.00	-0.01
GLN	220	-0.01	0.00	0.02	0.00	0.01	-0.01	0.00	0.00	0.00	-0.01
TYR	221	-0.03	-0.01	0.01	0.00	-0.03	-0.03	-0.01	0.03	0.00	-0.01
ASN	222	-0.06	-0.03	0.04	-0.01	-0.05	-0.06	-0.03	0.03	0.00	-0.05
HIE	223	-0.07	-0.03	0.07	0.00	-0.03	-0.07	-0.03	0.05	0.00	-0.04
ARG	224	-0.29	-0.47	0.62	-0.05	-0.19	-0.29	-0.47	0.74	0.00	-0.02
VAL	225	-0.07	-0.04	0.07	0.00	-0.04	-0.07	-0.04	0.07	0.00	-0.04
LYS	226	-0.15	-0.37	0.44	-0.03	-0.12	-0.15	-0.37	0.42	0.00	-0.11
LYS	227	-0.15	-0.18	0.23	-0.02	-0.13	-0.15	-0.18	0.27	0.00	-0.06
LEU	228	-0.12	-0.04	0.02	-0.01	-0.14	-0.12	-0.04	0.07	0.00	-0.09
ASN	229	-0.13	-0.17	0.22	-0.02	-0.11	-0.13	-0.17	0.26	0.00	-0.04
GLU	230	-0.07	-0.11	0.18	-0.01	-0.01	-0.07	-0.11	0.23	0.00	0.06
ILE	231	-0.02	0.00	0.02	0.00	0.00	-0.02	0.00	0.01	0.00	-0.01
SER	232	-0.03	-0.01	0.03	0.00	-0.01	-0.03	-0.01	0.02	0.00	-0.01
LYS	233	-0.03	0.09	0.00	0.00	0.05	-0.03	0.09	-0.08	0.00	-0.03
LEU	234	-0.01	0.02	-0.03	0.00	-0.02	-0.01	0.02	-0.03	0.00	-0.02
GLY	235	-0.02	0.00	0.00	0.00	-0.02	-0.02	0.00	0.01	0.00	-0.01
ILE	236	-0.03	-0.02	0.01	0.00	-0.03	-0.03	-0.02	0.02	0.00	-0.02
SER	237	-0.08	0.03	0.04	-0.01	-0.03	-0.08	0.03	0.03	0.00	-0.02
GLY	238	-0.03	-0.03	0.05	0.00	-0.01	-0.03	-0.03	0.05	0.00	-0.01
ASP	239	-0.01	0.17	-0.16	0.00	-0.01	-0.01	0.17	-0.17	0.00	-0.02
ILE	240	-0.01	0.00	0.01	0.00	0.00	-0.01	0.00	0.00	0.00	-0.01
ASP	241	0.00	0.05	-0.05	0.00	0.00	0.00	0.05	-0.04	0.00	0.01
LEU	242	0.00	0.00	0.01	0.00	0.00	0.00	0.00	0.00	0.00	0.00
THR	243	0.00	0.00	0.00	0.00	0.00	0.00	0.00	0.00	0.00	0.00
SER	244	0.00	0.00	0.00	0.00	0.00	0.00	0.00	0.00	0.00	0.00
ALA	245	0.00	0.00	0.01	0.00	0.00	0.00	0.00	0.00	0.00	0.00
SER	246	0.00	0.00	0.00	0.00	0.00	0.00	0.00	0.00	0.00	0.00
TYR	247	0.00	0.00	0.00	0.00	0.00	0.00	0.00	0.00	0.00	0.00
THR	248	0.00	0.00	0.00	0.00	0.00	0.00	0.00	0.00	0.00	0.00
MET	249	0.00	0.00	0.00	0.00	0.00	0.00	0.00	0.00	0.00	0.00
ILE	250	0.00	0.02	-0.02	0.00	0.00	0.00	0.02	-0.01	0.00	0.01

c) DSeDGal B binding mode

	Res	VDW	Eel	PB	NPOL	TOTAL	VDW	Eel	PB	NPOL	TOTAL
SES	111	-11.71	-12.89	17.84	-2.07	-8.84	-11.71	-12.89	11.99	0.00	-12.61
GLY	112	0.00	-0.04	0.06	0.00	0.02	0.00	-0.04	0.03	0.00	-0.02
PRO	113	-0.01	-0.01	0.01	0.00	-0.01	-0.01	-0.01	0.01	0.00	-0.01
LEU	114	0.00	0.00	0.00	0.00	0.00	0.00	0.00	0.00	0.00	0.00
ILE	115	0.00	0.00	0.01	0.00	0.00	0.00	0.00	0.00	0.00	0.00
VAL	116	-0.02	-0.01	0.01	0.00	-0.01	-0.02	-0.01	0.01	0.00	-0.02
PRO	117	-0.03	0.00	0.01	-0.01	-0.02	-0.03	0.00	0.00	0.00	-0.03
TYR	118	-0.01	-0.01	0.01	0.00	0.00	-0.01	-0.01	0.01	0.00	0.00
ASN	119	0.00	0.00	0.02	0.00	0.02	0.00	0.00	0.00	0.00	-0.01

LEU	120	0.00	0.00	0.01	0.00	0.00	0.00	0.00	0.00	0.00	0.00
PRO	121	-0.01	0.00	-0.01	0.00	-0.02	-0.01	0.00	0.00	0.00	-0.01
LEU	122	-0.01	-0.02	0.02	0.00	-0.02	-0.01	-0.02	0.02	0.00	-0.02
PRO	123	-0.02	0.00	0.01	0.00	-0.02	-0.02	0.00	0.01	0.00	-0.01
GLY	124	-0.02	-0.01	0.02	0.00	-0.02	-0.02	-0.01	0.03	0.00	-0.01
GLY	125	-0.02	0.00	0.00	0.00	-0.01	-0.02	0.00	-0.01	0.00	-0.03
VAL	126	0.00	0.00	0.01	0.00	0.00	0.00	0.00	0.00	0.00	0.00
VAL	127	0.00	0.00	0.00	0.00	0.00	0.00	0.00	0.00	0.00	0.00
PRO	128	0.00	0.00	0.00	0.00	0.00	0.00	0.00	0.00	0.00	0.00
ARG	129	0.00	0.00	0.00	0.00	0.00	0.00	0.00	-0.02	0.00	-0.02
MET	130	0.00	0.00	0.00	0.00	0.00	0.00	0.00	0.00	0.00	0.00
LEU	131	0.00	0.00	0.01	0.00	0.01	0.00	0.00	0.00	0.00	0.00
ILE	132	0.00	0.00	0.01	0.00	0.00	0.00	0.00	0.00	0.00	0.00
THR	133	-0.01	-0.01	0.03	0.00	0.00	-0.01	-0.01	0.01	0.00	-0.01
ILE	134	-0.02	0.01	-0.02	0.00	-0.03	-0.02	0.01	-0.01	0.00	-0.02
LEU	135	-0.25	-0.04	0.00	-0.04	-0.33	-0.25	-0.04	0.03	0.00	-0.27
GLY	136	-0.11	-0.01	0.02	0.00	-0.10	-0.11	-0.01	0.09	0.00	-0.03
THR	137	-0.08	-0.02	0.06	-0.01	-0.04	-0.08	-0.02	0.05	0.00	-0.05
VAL	138	-0.02	-0.01	0.03	0.00	0.00	-0.02	-0.01	0.01	0.00	-0.02
LYS	139	-0.01	-0.07	0.10	0.00	0.02	-0.01	-0.07	0.07	0.00	-0.02
PRO	140	0.00	0.00	0.00	0.00	0.00	0.00	0.00	0.00	0.00	0.00
ASN	141	0.00	-0.01	0.02	0.00	0.01	0.00	-0.01	0.01	0.00	0.00
ALA	142	-0.01	0.00	0.02	0.00	0.01	-0.01	0.00	0.01	0.00	0.00
ASN	143	-0.06	0.05	0.01	-0.01	-0.01	-0.06	0.05	0.00	0.00	-0.01
ARG	144	-0.49	-0.54	0.82	-0.10	-0.31	-0.49	-0.54	1.02	0.00	-0.01
ILE	145	-0.10	0.10	-0.11	0.00	-0.12	-0.10	0.10	-0.14	0.00	-0.14
ALA	146	-0.21	-0.04	0.01	-0.03	-0.27	-0.21	-0.04	0.03	0.00	-0.22
LEU	147	-0.05	0.04	-0.01	0.00	-0.03	-0.05	0.04	-0.03	0.00	-0.05
ASP	148	-0.08	-0.42	0.60	-0.01	0.09	-0.08	-0.42	1.01	0.00	0.51
PHE	149	-0.01	0.00	-0.01	0.00	-0.02	-0.01	0.00	0.00	0.00	-0.01
GLN	150	-0.02	0.02	-0.01	0.00	-0.01	-0.02	0.02	-0.04	0.00	-0.04
ARG	151	-0.13	-0.08	0.12	-0.01	-0.10	-0.13	-0.08	0.03	0.00	-0.18
GLY	152	-0.07	-0.04	0.05	-0.01	-0.07	-0.07	-0.04	0.06	0.00	-0.05
ASN	153	-0.04	-0.05	0.07	-0.01	-0.03	-0.04	-0.05	0.06	0.00	-0.03
ASP	154	-0.03	-0.13	0.15	0.00	-0.01	-0.03	-0.13	0.31	0.00	0.15
VAL	155	-0.06	0.01	-0.03	0.00	-0.08	-0.06	0.01	-0.01	0.00	-0.06
ALA	156	-0.01	0.01	0.01	0.00	0.01	-0.01	0.01	-0.01	0.00	-0.01
PHE	157	-0.02	-0.01	-0.02	0.00	-0.06	-0.02	-0.01	0.03	0.00	0.00
HIE	158	-0.84	-0.54	0.74	-0.10	-0.75	-0.84	-0.54	1.14	0.00	-0.24
PHE	159	-0.07	0.02	-0.09	0.00	-0.14	-0.07	0.02	-0.02	0.00	-0.08
ASN	160	-0.68	-0.12	-0.02	-0.05	-0.87	-0.68	-0.12	0.17	0.00	-0.63
PRO	161	-0.05	0.08	0.00	0.00	0.04	-0.05	0.08	-0.12	0.00	-0.09
ARG	162	-0.98	-2.37	2.55	-0.18	-0.98	-0.98	-2.37	2.63	0.00	-0.72
PHE	163	-0.02	-0.02	0.03	0.00	-0.02	-0.02	-0.02	0.04	0.00	-0.01
ASN	164	-0.03	-0.03	0.07	0.00	0.01	-0.03	-0.03	0.07	0.00	0.01
GLU	165	-0.11	0.26	-0.23	-0.01	-0.09	-0.11	0.26	-0.19	0.00	-0.03
ASN	166	-0.01	0.00	0.02	0.00	0.01	-0.01	0.00	0.00	0.00	-0.01
ASN	167	-0.01	-0.01	0.03	0.00	0.01	-0.01	-0.01	0.02	0.00	0.00
ARG	168	-0.02	-0.11	0.16	0.00	0.03	-0.02	-0.11	0.10	0.00	-0.03
ARG	169	-0.03	-0.19	0.24	0.00	0.02	-0.03	-0.19	0.20	0.00	-0.01
VAL	170	-0.02	0.03	-0.05	0.00	-0.03	-0.02	0.03	-0.05	0.00	-0.03

ILE	171	-0.02	-0.03	-0.02	0.00	-0.07	-0.02	-0.03	0.04	0.00	-0.01
VAL	172	-0.39	-0.02	-0.08	-0.02	-0.52	-0.39	-0.02	0.21	0.00	-0.20
CYS	173	-0.14	0.03	-0.11	0.00	-0.22	-0.14	0.03	0.07	0.00	-0.04
ASN	174	-0.32	-1.12	0.33	-0.04	-1.15	-0.32	-1.12	0.90	0.00	-0.54
THR	175	-0.06	0.05	-0.06	0.00	-0.07	-0.06	0.05	-0.06	0.00	-0.07
LYS	176	-0.10	0.10	0.08	0.00	0.07	-0.10	0.10	-0.01	0.00	-0.01
LEU	177	-0.01	0.00	0.01	0.00	0.00	-0.01	0.00	-0.01	0.00	-0.02
ASP	178	-0.01	-0.05	0.07	0.00	0.02	-0.01	-0.05	0.10	0.00	0.04
ASN	179	-0.02	-0.01	0.06	0.00	0.03	-0.02	-0.01	0.02	0.00	-0.02
ASN	180	-0.04	-0.01	0.05	0.00	0.00	-0.04	-0.01	0.02	0.00	-0.03
TRP	181	-1.81	-0.48	0.46	-0.23	-2.06	-1.81	-0.48	0.59	0.00	-1.70
GLY	182	-0.07	0.02	0.04	0.00	-0.01	-0.07	0.02	0.02	0.00	-0.03
ARG	183	-0.05	-0.05	0.04	0.00	-0.06	-0.05	-0.05	0.03	0.00	-0.07
GLU	184	-0.17	-2.57	2.69	-0.07	-0.12	-0.17	-2.57	3.78	0.00	1.04
GLU	185	-0.03	0.13	-0.12	0.00	-0.02	-0.03	0.13	-0.16	0.00	-0.06
ARG	186	-0.08	-0.10	0.21	0.00	0.03	-0.08	-0.10	0.10	0.00	-0.07
GLN	187	-0.01	0.01	0.01	0.00	0.01	-0.01	0.01	-0.01	0.00	-0.01
SER	188	-0.01	0.01	0.00	0.00	0.00	-0.01	0.01	0.00	0.00	-0.01
VAL	189	-0.05	0.00	0.01	-0.01	-0.05	-0.05	0.00	0.00	0.00	-0.05
PHE	190	-0.05	-0.04	0.05	-0.01	-0.05	-0.05	-0.04	0.08	0.00	-0.01
PRO	191	-0.12	-0.24	0.24	-0.02	-0.15	-0.12	-0.24	0.17	0.00	-0.18
PHE	192	-0.07	-0.02	0.02	0.00	-0.07	-0.07	-0.02	0.08	0.00	-0.01
GLU	193	-0.08	0.15	-0.12	-0.01	-0.06	-0.08	0.15	-0.12	0.00	-0.05
SER	194	-0.01	0.01	0.01	0.00	0.01	-0.01	0.01	-0.01	0.00	-0.01
GLY	195	-0.01	0.00	0.03	0.00	0.01	-0.01	0.00	0.00	0.00	-0.01
LYS	196	-0.22	-0.53	0.64	-0.04	-0.14	-0.22	-0.53	0.63	0.00	-0.12
PRO	197	-0.56	-0.25	0.27	-0.10	-0.64	-0.56	-0.25	0.36	0.00	-0.45
PHE	198	-0.34	-0.14	0.12	-0.02	-0.37	-0.34	-0.14	0.19	0.00	-0.29
LYS	199	-0.50	-0.02	0.23	-0.05	-0.34	-0.50	-0.02	0.14	0.00	-0.38
ILE	200	-0.02	0.01	0.00	0.00	-0.01	-0.02	0.01	-0.01	0.00	-0.03
GLN	201	-0.02	-0.01	0.04	0.00	0.01	-0.02	-0.01	0.01	0.00	-0.02
VAL	202	0.00	0.00	0.01	0.00	0.01	0.00	0.00	0.00	0.00	0.00
LEU	203	0.00	0.01	0.00	0.00	0.00	0.00	0.01	-0.01	0.00	0.00
VAL	204	0.00	0.00	0.01	0.00	0.01	0.00	0.00	0.00	0.00	0.00
GLU	205	0.00	0.01	-0.01	0.00	0.00	0.00	0.01	0.02	0.00	0.03
PRO	206	0.00	0.00	0.00	0.00	0.00	0.00	0.00	0.00	0.00	0.00
ASP	207	0.00	0.03	-0.03	0.00	0.00	0.00	0.03	0.00	0.00	0.03
HIE	208	0.00	0.00	0.01	0.00	0.01	0.00	0.00	0.00	0.00	0.00
PHE	209	0.00	0.00	-0.01	0.00	-0.01	0.00	0.00	0.00	0.00	-0.01
LYS	210	0.00	-0.01	0.02	0.00	0.01	0.00	-0.01	-0.02	0.00	-0.03
VAL	211	-0.01	-0.01	0.03	0.00	0.01	-0.01	-0.01	0.01	0.00	-0.01
ALA	212	-0.01	0.03	-0.02	0.00	-0.01	-0.01	0.03	-0.03	0.00	-0.01
VAL	213	-0.06	-0.08	0.06	0.00	-0.08	-0.06	-0.08	0.09	0.00	-0.04
ASN	214	-0.63	-0.28	0.38	-0.08	-0.62	-0.63	-0.28	0.53	0.00	-0.38
ASP	215	0.01	-2.39	2.20	-0.04	-0.23	0.01	-2.39	2.53	0.00	0.14
ALA	216	-0.04	-0.01	0.02	-0.01	-0.04	-0.04	-0.01	0.01	0.00	-0.04
HIE	217	-0.02	-0.08	0.09	0.00	-0.01	-0.02	-0.08	0.08	0.00	-0.02
LEU	218	-0.04	-0.01	0.03	0.00	-0.03	-0.04	-0.01	0.01	0.00	-0.04
LEU	219	-0.01	-0.01	0.02	0.00	0.00	-0.01	-0.01	0.01	0.00	0.00
GLN	220	0.00	0.00	0.01	0.00	0.01	0.00	0.00	0.00	0.00	0.00
TYR	221	-0.01	0.01	-0.03	0.00	-0.04	-0.01	0.01	-0.01	0.00	-0.02

ASN	222	0.00	0.01	0.00	0.00	0.01	0.00	0.01	-0.01	0.00	0.00
HIE	223	0.00	0.00	0.00	0.00	0.01	0.00	0.00	0.00	0.00	0.00
ARG	224	-0.01	-0.23	0.34	0.00	0.10	-0.01	-0.23	0.21	0.00	-0.03
VAL	225	0.00	0.00	0.01	0.00	0.01	0.00	0.00	0.00	0.00	0.00
LYS	226	0.00	-0.04	0.04	0.00	0.01	0.00	-0.04	0.01	0.00	-0.03
LYS	227	-0.01	-0.02	0.04	0.00	0.01	-0.01	-0.02	0.00	0.00	-0.03
LEU	228	0.00	0.00	0.00	0.00	0.00	0.00	0.00	0.01	0.00	0.00
ASN	229	-0.02	-0.03	0.04	0.00	-0.02	-0.02	-0.03	0.07	0.00	0.01
GLU	230	-0.10	-0.08	0.10	-0.02	-0.09	-0.10	-0.08	0.24	0.00	0.06
ILE	231	-0.02	-0.01	0.01	0.00	-0.02	-0.02	-0.01	0.02	0.00	-0.01
SER	232	-0.06	-0.05	0.07	-0.01	-0.05	-0.06	-0.05	0.08	0.00	-0.02
LYS	233	-0.02	0.02	0.02	0.00	0.03	-0.02	0.02	-0.03	0.00	-0.03
LEU	234	-0.01	0.00	0.00	0.00	-0.01	-0.01	0.00	-0.01	0.00	-0.01
GLY	235	-0.01	0.02	-0.02	0.00	-0.01	-0.01	0.02	-0.01	0.00	0.00
ILE	236	-0.02	-0.02	0.02	0.00	-0.02	-0.02	-0.02	0.03	0.00	-0.01
SER	237	-0.10	-0.04	0.10	-0.02	-0.07	-0.10	-0.04	0.11	0.00	-0.04
GLY	238	-0.06	-0.03	0.07	-0.01	-0.03	-0.06	-0.03	0.08	0.00	-0.01
ASP	239	-0.02	-0.11	0.09	-0.01	-0.05	-0.02	-0.11	0.18	0.00	0.05
ILE	240	-0.01	-0.01	0.02	0.00	0.00	-0.01	-0.01	0.01	0.00	-0.02
ASP	241	-0.01	0.09	-0.09	0.00	-0.02	-0.01	0.09	-0.07	0.00	0.01
LEU	242	-0.02	-0.02	0.02	0.00	-0.02	-0.02	-0.02	0.02	0.00	-0.02
THR	243	-0.10	-0.09	0.13	-0.02	-0.08	-0.10	-0.09	0.13	0.00	-0.06
SER	244	-0.01	0.01	0.00	0.00	0.00	-0.01	0.01	-0.01	0.00	-0.01
ALA	245	0.00	0.00	0.01	0.00	0.01	0.00	0.00	0.00	0.00	0.00
SER	246	0.00	0.00	0.00	0.00	0.00	0.00	0.00	0.00	0.00	0.00
TYR	247	0.00	0.00	0.00	0.00	0.00	0.00	0.00	0.00	0.00	0.00
THR	248	0.00	0.00	0.00	0.00	0.00	0.00	0.00	0.00	0.00	0.00
MET	249	0.00	0.00	0.00	0.00	0.00	0.00	0.00	0.00	0.00	0.00
ILE	250	0.00	0.00	0.00	0.00	0.00	0.00	0.00	0.02	0.00	0.02

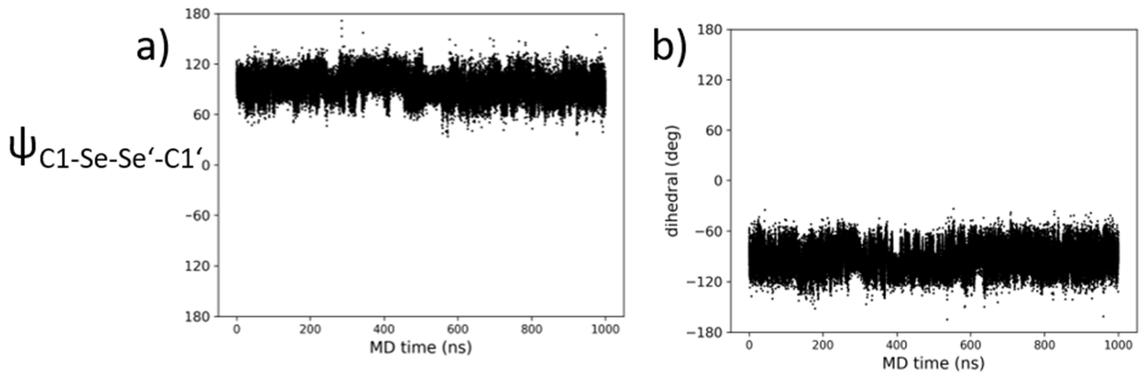

Figure S4. The evolution of the C1-Se-Se-C1′ central dihedral angle in the DSeDGal – *h*Gal3 complex in the MD simulation starting from binding mode A (a) and in B (b).
